# Efficacy and safety of PARP inhibitors combined with antiangiogenic agents in the maintenance treatment of ovarian cancer: a systematic review and meta-analysis with trial sequential analysis of randomized controlled trials

**DOI:** 10.3389/fphar.2024.1372077

**Published:** 2024-03-22

**Authors:** Yan Wei, Li He, Tao Liu, Tao Guo, Cong Xie, Jigang Jia, Yonghong Lin, Jiang Liu, Jiayin Fan

**Affiliations:** ^1^ Department of Gynecology, Chengdu Women’s and Children’s Central Hospital, School of Medicine, University of Electronic Science and Technology of China, Chengdu, China; ^2^ Department of Obstetrics and Gynecology, West China Second University Hospital, Sichuan University, Chengdu, China

**Keywords:** PARP inhibitors, antiangiogenic agents, olaparib, bevacizumab, ovarian cancer, combination therapy, meta-analysis

## Abstract

**Background:** Poly (ADP-ribose) polymerase (PARP) inhibitor and antiangiogenic agent monotherapy have shown to be effective as maintenance treatment in patients with ovarian cancer (OC). However, there is currently a lack of evidence-based study to directly compare the effects of combination therapy with these two drugs. Therefore, this study aimed to compare the efficacy and safety of combination therapy with PARP inhibitors and antiangiogenic agents in women with OC using a meta-analysis.

**Methods:** An exhaustive search of literature was undertaken using multiple databases, including PubMed, Web of Science, Embase, and the Cochrane Library to identify pertinent randomized controlled trials (RCTs) published up until 17 December 2023. The data on progression-free survival (PFS), overall survival (OS), and adverse events (AEs) were pooled. We computed the pooled hazard ratios (HRs) and their 95% confidence intervals (CIs) for PFS and OS, along with the relative risks (RRs) and 95% CIs for AEs. Trial sequential analysis, heterogeneity test, sensitivity analysis, and publication bias assessment were performed. Stata 12.0 and Software R 4.3.1 were utilized for all analyses.

**Results:** This meta-analysis included 7 RCTs with a total of 3,388 participants. The overall analysis revealed that combination therapy of PARP inhibitors and antiangiogenic agents significantly improved PFS (HR = 0.615, 95% CI = 0.517–0.731; 95% PI = 0.379–0.999), but also increased the risk of AEs, including urinary tract infection (RR = 1.500, 95% CI = 1.114–2.021; 95% PI = 0.218–10.346), fatigue (RR = 1.264, 95% CI = 1.141–1.400; 95% PI = 1.012–1.552), headache (RR = 1.868, 95% CI = 1.036–3.369; 95% PI = 0.154–22.642), anorexia (RR = 1.718, 95% CI = 1.320–2.235; 95% PI = 0.050–65.480), and hypertension (RR = 5.009, 95% CI = 1.103–22.744; 95% PI = 0.016–1580.021) compared with PARP inhibitor or antiangiogenic agent monotherapy. Our study has not yet confirmed the benefit of combination therapy on OS in OC patients (HR = 0.885, 95% CI = 0.737–1.063). Additionally, subgroup analyses further showed that combination therapy resulted in an increased risk of AEs, encompassing thrombocytopenia, vomiting, abdominal pain, proteinuria, fatigue, headache, anorexia, and hypertension (all *p* < 0.05).

**Conclusion:** Our study demonstrated the PFS benefit of combination therapy with PARP inhibitors and antiangiogenic agents in patients with OC. The OS result need to be updated after the original trial data is mature. Clinicians should be vigilant of AEs when administering the combination therapy in clinical practice.

**Systematic Review Registration:** https://www.crd.york.ac.uk/PROSPERO/, identifier CRD42023494482.

## 1 Introduction

Ovarian cancer (OC) is a prevalent gynecologic malignancy and the leading cause of mortality among females facing gynecological malignancies (Siegel et al., 2020). Given the difficulty in detecting OC during its early stages, a significant number of patients receive their diagnosis at an advanced stage, leading to a reduced 5-year relative survival rate (Wang et al., 2021). Treatment for advanced OC typically involves cytoreductive surgery and platinum-based chemotherapy. However, despite its initial efficacy, approximately 70% of patients experience a recurrence post-primary treatment, gravely impacting survival duration (Giornelli, 2016; Capriglione et al., 2017; Coleridge et al., 2021). Researches have indicated the efficacy of maintenance chemotherapy in extending remission periods (Markman et al., 2003; Markman et al., 2009; Abaid et al., 2010). Presently, novel targeted treatments are being explored to manage OC and prevent its recurrence. Foremost among these are poly (ADP-ribose) polymerase (PARP) inhibitors and antiangiogenic agents.

PARP inhibitors have surfaced as a notable category of drugs for women experiencing recurrent OC in various contexts, such as treating BRCA mutation-associated relapsed conditions or as maintenance therapy in platinum-sensitive cases after responding to platinum-based treatments (Liu et al., 2019). PARP inhibitors have demonstrated their ability to induce DNA damage through the catalytic inhibition of PARP enzyme and entrapping DNA-PARP complexes, fostering synthetic lethality in cells impaired in homologous recombination repair, thereby enhancing the destruction of tumor cells (Ding et al., 2018; O'Sullivan et al., 2014). Currently, multiple PARP inhibitors (e.g., olaparib, niraparib, rucaparib, veliparib, and talazoparib) are undergoing trials in different phases of development, either in combination with other drug categories or as a standalone agent (Hopkins et al., 2019). The pairing of PARP inhibitors with antiangiogenic agents is a growing area of interest in OC research. Antiangiogenic medications hinder tumor vascularization and impede tumor cells from accessing nutrients by inflicting damage on established tumor blood vessels and obstructing the formation of new ones (Abdalla et al., 2018; Jászai and Schmidt, 2019). As a result, antiangiogenic agents have evolved into a promising drug class for OC patients. Furthermore, the potential for therapeutic synergy is particularly notable when combining PARP inhibitors with antiangiogenic agents. The hypoxia triggered by antiangiogenic treatments may escalate DNA damage and genetic instability (Chan and Bristow, 2010a), culminating in defective homologous recombination that could heighten sensitivity to PARP inhibitors (Hegan et al., 2010).

Although several high-quality randomized, phase II/III trials in recent years have shown that maintenance combination therapy with PARP inhibitors (olaparib or niraparib) and antiangiogenic agents (bevacizumab or cediranib) significantly improved progression-free survival (PFS) *versus* PARP inhibitor or antiangiogenic agent monotherapy after first-line treatment for OC (Liu et al., 2019; Mirza et al., 2019; Ray-Coquard et al., 2023), the conclusions derived from the randomized controlled trials (RCTs) remain a subject of debate (Vergote et al., 2021; Liu et al., 2022). Moreover, combination therapy might be more susceptible to adverse events (AEs) compared to monotherapy (Ray-Coquard et al., 2019). Consequently, this study conducted a systematic review and meta-analysis of RCTs to determine the clinical efficacy and safety of maintenance combination therapy of PARP inhibitors and antiangiogenic agents *versus* PARP inhibitor or antiangiogenic agent monotherapy in patients with OC.

## 2 Materials and methods

### 2.1 Study design

In compliance with the Preferred Reporting Items for Systematic Reviews and Meta-Analysis (PRISMA) guidelines, this meta-analysis was carried out (Page et al., 2021). Concurrently, the protocol for this study was registered in anticipation with the PROSPERO database, under the identifier CRD42023494482.

### 2.2 Literature search strategy

We undertook a comprehensive search of databases such as PubMed, Web of Science, Embase, and the Cochrane Library for pertinent studies published prior to 17 December 2023. The primary search treatment-related retrieval fields included: “angiogenesis inhibitors”, “tyrosine kinases inhibitors”, “bevacizumab”, “cediranib”, “recentin”, “avastin”, “aflibercept”, “votrient”, “sunitinib” AND “PARP inhibitors”, “olaparib”, “lynparza”, “rucaparib”, “talazoparib”, “niraparib”, “veliparib”, “rubraca”, “talzenna”. The cancer-related retrieval fields included: “ovarian cancer”, “ovary cancer”, “ovarian neoplasm”, “cancer of ovary”. No additional restrictions were imposed, encompassing language. Furthermore, to uncover more pertinent studies, we also scoured the reference lists of all relevant review articles. A detailed search strategy was presented in Supplementary Material S1.

### 2.3 Inclusion and exclusion criteria

The selection process for relevant literature involved a rigorous screening protocol based on the following inclusion criteria: (i) RCTs; (ii) patients must have a histologically or cytologically confirmed diagnosis of ovarian, primary peritoneal, or fallopian tube cancer; (iii) intervention: PARP inhibitors plus antiangiogenic agents; (iv) comparison: PARP inhibitors or antiangiogenic agents as a single agent; (v) outcomes: PFS, overall survival (OS), or AEs. Studies were excluded if they (i) were not RCTs; (ii) failed to report on the outcomes of interest; (iii) included trial populations with overlaps; (iv) were case reports, editorial comments, animal studies, conference abstracts, or reviews.

### 2.4 Data extraction and endpoint

Two independent reviewers conducted the data extraction process, with any discrepancies in study eligibility being settled through mutual agreement. We collated the following information from the selected studies: first author’s name, publication year, abbreviation of RCT, trial phase, disease setting, treatment line, regimen details in experimental and control arm, number and age of patients allocated for each arm, follow-up duration, and outcomes. The primary endpoints for this meta-analysis were PFS and OS, while secondary endpoints included AEs like fatigue, hypertension, and nausea. In cases where multiple publications reported results from the same trial, we prioritized the most recent or comprehensive publication that provided the relevant information. For studies where PFS or OS data could not be directly extracted, we utilized Engauge Digitizer 10.8 (http://markummitchell.github.io/engauge-digitizer/) and the methodology proposed by Tierney et al. (Tierney et al., 2007) to extract data from the Kaplan-Meier curves.

### 2.5 Risk of bias assessment

The assessment of the included RCTs’ quality was conducted using the modified Jadad scale (Jadad et al., 1996). Each study was independently appraised by two reviewers on aspects, including the randomization procedure, concealment of allocation, implementation of double-blinding, and the reporting of withdrawals and dropouts. Any divergences in assessment were settled through consensus. Trials were scored and classified as either high quality (4-7 points) or low quality (0–3 points).

### 2.6 Statistical analysis

We computed the pooled hazard ratios (HRs) and their 95% confidence intervals (CIs) for PFS and OS, along with the relative risks (RRs) and 95% CIs for AEs. HR (or RR) > 1 was interpreted as favoring the control group, whereas HR (or RR) < 1 indicated preference for the intervention group. To assess the heterogeneity across studies, we employed the Cochrane Q-test, I^2^ statistics, and 95% prediction interval (PI) (Bowden et al., 2011; IntHout et al., 2016). Based on these heterogeneity outcomes, we applied either the Mantel-Haenszel fixed-effects model or the DerSimonian-Laird random-effects model to derive the pooled effects. The threshold for employing a random-effects model was set at I^2^ > 50% or *p*-value <0.10, indicating moderate to high heterogeneity; otherwise, a fixed-effects model was utilized (Higgins and Thompson, 2002). We performed subgroup analysis based on specific PARP inhibitors and antiangiogenic drugs. Publication bias was assessed through funnel plots and Begg’s and Egger’s tests (Begg and Mazumdar, 1994; Egger et al., 1997), with the trim-and-fill method adjusting for any detected bias (Duval and Tweedie, 2000). We conducted a sensitivity analysis by excluding each study in turn to assess changes in the combined HR or RR. All statistical analyses were carried out using R software 4.3.1 and Stata 12.0 (Stata Corp. College Station, Texas, United States). A two-sided *p* < 0.05 was considered statistically significant.

### 2.7 Trial sequential analysis

In our pursuit to rigorously evaluate the efficacy and safety of the combination of PARP inhibitors with antiangiogenic agents in OC patients, we employed trial sequential analysis (TSA). This methodology was applied to PFS and OS data using Stata software version 12.0 and R software version 4.3.1, while AEs were scrutinized using TSA software version 0.9.5.10 Beta (www.ctu.dk/tsa). TSA aimed to determine whether the current data suffices for a conclusive evidence base, known as the required information size (RIS) (Wetterslev et al., 2017). We utilized the “metacumbounds” and “rsource” functions within Stata 12.0, and the “foreign” and “ldbounds” packages in R software to conduct TSA for PFS and OS, adopting an *a priori* information size (APIS) approach (Xie et al., 2022). For the analysis of AEs, the TSA software was harnessed to calculate the RIS and establish the O’Brien-Fleming α-spending boundaries, adhering to 5% type I error and 20% type II error, both set as two-sided thresholds. A crossing of the cumulative Z-curve over the RIS or the trial sequential monitoring boundary signaled that additional studies were redundant, providing substantial evidence to either support or reject the effect of intervention.

## 3 Results

### 3.1 Study selection

**FIGURE 1:**
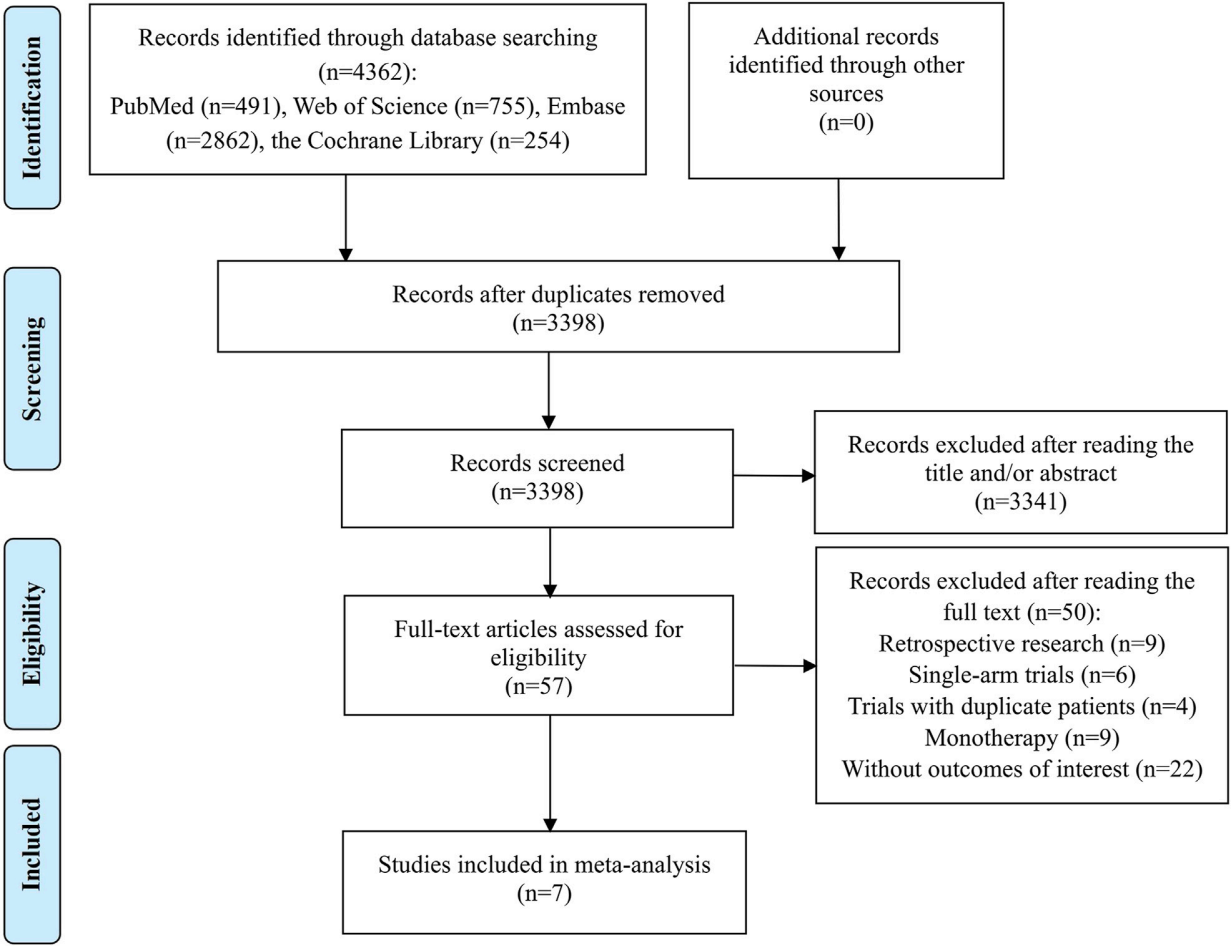
Flow diagram of the process of study selection.

The preliminary search identified 4,362 records, from which 964 were discarded as duplicates. The subsequent step involved a careful review of the titles and abstracts of the remaining 3,398 studies, leading to the elimination of 3,341 papers that did not align with our research topic. Of the remaining 57 studies deemed potentially relevant, a full-text review was conducted, resulting in the exclusion of 50 studies for the following reasons: 9 were retrospective research; 6 were single-arm trials; 4 trials contained duplicate patients; 9 studies focused solely on monotherapy for OC; and 22 articles did not provide the required outcome data. Ultimately, 7 studies met the criteria and were included in the meta-analysis (Liu et al., 2019; Mirza et al., 2019; Ray-Coquard et al., 2019; Vergote et al., 2021; Liu et al., 2022; Ray-Coquard et al., 2023; Sabatier et al., 2023). The process of study identification and selection was depicted in Figure 1.

### 3.2 Characteristics and quality assessment of included studies

The characteristics of these included 7 RCTs (2 phase II trials and 5 phase III trials) were shown in Table 1. The research articles were published from 2019 to 2023 in English. The interventions in each study were maintenance therapies administered to OC patients following first-line treatment. A total of 2,043 OC patients were assigned to a combination of PARP inhibitors and antiangiogenic agents, whereas 1,345 patients received either PARP inhibitors alone or antiangiogenic agents with placebo. 4 trials investigated the combination therapy of olaparib and bevacizumab, 2 trials examined the pairing of olaparib and cediranib, and one study specifically explored the combination of niraparib and bevacizumab. All studies included in this analysis were deemed to be of high quality. A significant methodological shortcoming observed was the absence of double blinding in the trial design. More information on the quality assessment can be located in Supplementary Material S2.

**TABLE 1:** Characteristics of the included RCTs.

First author (Year)	Trial	Study phase	Disease setting	Line	Sample size (E/C)	Age [median (range), years]	Experimental arm	Control arm	Median duration of follow-up (months)	Meta-analysis end-points
Sabatier et al. (2023)	PAOLA-1/ENGOT-ov25	III	Newly diagnosed advanced, high-grade ovarian cancer	Maintenance after first-line platinum-taxane-bevacizumab triplet treatment	537/269	26–87	Olaparib 300 mg twice daily + Bevacizumab	Placebo twice daily + Bevacizumab	22.1	AEs
Ray-Coquard et al. (2023)	PAOLA-1/ENGOT-ov25	III	Newly diagnosed advanced stage, high-grade serous or endometrioid ovarian cancer	Maintenance after first-line platinum-based chemotherapy plus bevacizumab treatment	537/269	E: 61 (32–87); C: 60 (26–85)	Olaparib 300 mg twice daily + Bevacizumab	Placebo twice daily + Bevacizumab	E: 61.7; C: 61.9	PFS, OS
Liu et al. (2022)	NRG-GY004	III	Platinum-sensitive relapsed high-grade serous or high-grade endometrioid ovarian, primary peritoneal, or fallopian tube cancer	Maintenance after first-line platinum-based chemotherapy	189/189	>18 years	Olaparib 200 mg tablets twice daily + Cediranib 30 mg tablet once daily	Olaparib 300 mg tablets twice daily	24 (Mean)	PFS, AEs
Liu et al. (2019)	NCT01116648	II	Relapsed platinum-sensitive ovarian cancer	Maintenance after anti-angiogenic therapy in the first-line setting	44/46	E: 57.8 (41.9–85.6); C: 58.1 (32.7–81.9)	Cediranib 30 mg orally daily + Olaparib capsules 200 mg orally twice daily	Olaparib capsule monotherapy 400 mg orally twice daily	46	PFS, OS, AEs
Vergote et al. (2021)	Pooled analysis of SOLO1 and PAOLA-1/ENGOT-ov25	III	Newly diagnosed, advanced BRCA-mutated ovarian cancer	Maintenance after first-line treatment with platinum-based chemotherapy or platinum-based chemotherapy plus bevacizumab	151/254	E: 54.3 (mean age); C: 53.6 (mean age)	Olaparib 300 mg twice daily + Bevacizumab	Olaparib 300 mg twice daily	E: 22.7; C: 40.7	PFS, AEs
Mirza et al. (2019)	NSGO-AVANOVA2/ENGOT-ov24	II	Platinum-sensitive recurrent ovarian cancer	Maintenance after first-line platinum-based chemotherapy	48/49	E: 67 (IQR: 59–70); C: 66 (IQR: 58–70)	Niraparib 300 mg once daily + Bevacizumab 15 mg/kg every 3 weeks	Niraparib 300 mg once daily	16.9	PFS, AEs
Ray-Coquard et al. (2019)	PAOLA-1	III	Newly diagnosed advanced, high-grade serous or endometrioid ovarian cancer, primary peritoneal cancer, or fallopian-tube cancer	Maintenance after first-line platinum-taxane chemotherapy plus bevacizumab treatment	537/269	E: 61 (32–87); C: 60 (26–85)	Olaparib 300 mg twice daily + Bevacizumab	Placebo twice daily + Bevacizumab	22.9	PFS, AEs

E, Experimental group; C, Control group; PFS, progression-free survival; AEs, adverse events; OS, overall survival; IQR: interquartile range.

### 3.3 Meta-analysis of efficacy outcomes

**FIGURE 2:**
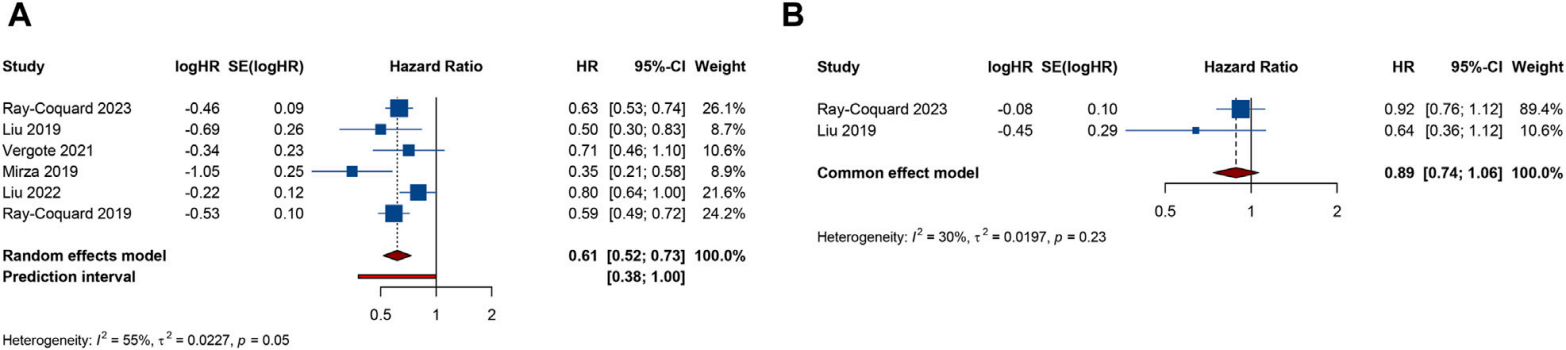
Forest plot of efficacy outcomes after combination therapy with PARP inhibitors and antiangiogenic drugs for ovarian cancer. **(A)** progression-free survival; **(B)** overall survival.

6 RCTs analyzed PFS outcome. The trials demonstrated significant heterogeneity (I^2^ = 54.8%, Tau^2^ = 0.0227), prompting the adoption of a random-effects model for analysis. The results revealed that combination therapy with PARP inhibitors and antiangiogenic drugs resulted in a significantly better pooled PFS than PARP inhibitor or antiangiogenic monotherapy (HR = 0.615, 95% CI = 0.517–0.731; 95% PI = 0.379–0.999) (Table 2; Figure 2A). Subgroup analysis based on the specific drugs of PARP inhibitors and antiangiogenic therapy showed that the combination therapy of olaparib and bevacizumab yielded a significant PFS benefit (HR = 0.613, 95% CI = 0.540–0.695; I^2^ = 0%, Tau^2^ = 0) over bevacizumab monotherapy (Table 3; Supplementary Figure S1).

**TABLE 2:** Pooled effect of the efficacy and safety outcomes of PARP inhibitors combined with antiangiogenic agents for ovarian cancer.

Outcomes	Number of studies	Meta-analysis				Heterogeneity	
		HR/RR	95% CI	*p*-value	95% PI	I^2^, Tau^2^	*p*-Value
PFS	6	0.615	0.517–0.731	<0.001	0.379–0.999	54.8%, 0.0227	0.050
OS	2	0.885	0.737–1.063	0.193	-	29.9%, 0.0197	0.232
Anemia	5	1.106	0.490–2.498	0.809	0.048–25.480	95.5%, 0.7989	<0.001
Leukopenia	3	1.293	0.732–2.285	0.376	0.002–712.363	68.9%, 0.1624	0.040
Neutropenia	5	1.054	0.833–1.332	0.662	0.706–1.597	1.6%, 0.0014	0.397
Thrombocytopenia	5	1.427	0.832–2.449	0.197	0.248–8.227	62.7%, 0.2271	0.030
Nausea	5	1.210	0.818–1.788	0.340	0.268–5.459	94.7%, 0.1845	<0.001
Vomiting	5	1.264	0.756–2.115	0.372	0.196–8.139	86.4%, 0.2735	<0.001
Diarrhea	5	1.757	0.739–4.177	0.202	0.073–42.406	94.7%, 0.8056	<0.001
Abdominal pain	4	1.156	0.952–1.402	0.143	0.364–3.778	46.2%, 0.0456	0.134
Constipation	4	0.976	0.791–1.204	0.822	0.410–2.347	27.3%, 0.0217	0.248
Urinary tract infection	3	1.500	1.114–2.021	0.008	0.218–10.346	0%, 0	0.981
Proteinuria	4	7.195	0.235–219.980	0.258	-	90.7%, 10.6739	<0.001
Fatigue	4	1.264	1.141–1.400	<0.001	1.012–1.552	0%, 0	0.671
Headache	4	1.868	1.036–3.369	0.038	0.154–22.642	76.0%, 0.2457	0.006
Anorexia	3	1.718	1.320–2.235	<0.001	0.050–65.480	22.3%, 0.0382	0.276
Dyspnea	3	1.272	0.928–1.742	0.135	0.004–443.951	34.8%, 0.1164	0.216
Hypertension	5	5.009	1.103–22.744	0.037	0.016–1580.021	98.2%, 2.6730	<0.001

**TABLE 3:** Subgroup analysis of the efficacy and safety outcomes of PARP inhibitors combined with antiangiogenic agents for ovarian cancer.

Outcomes and subgroups	Number of studies	Meta-analysis			Heterogeneity	
		HR/RR	95% CI	*p*-value	I2, Tau2	*p*-Value
PFS						
Olaparib plus Bevacizumab vs. Bevacizumab	2	0.613	0.540–0.695	<0.001	0%, 0	0.614
Cediranib plus Olaparib vs. Olaparib	2	0.670	0.429–1.047	0.079	63.4%, 0.0700	0.099
Bevacizumab plus Olaparib (or Niraparib) vs. Olaparib (or Niraparib)	2	0.503	0.252–1.007	0.052	76.9%, 0.1922	0.038
Anemia						
Cediranib plus Olaparib vs. Olaparib	2	0.538	0.249–1.163	0.115	63.0%, 0.2126	0.100
Bevacizumab plus Olaparib (or Niraparib) vs. Olaparib (or Niraparib)	2	1.053	0.844–1.314	0.647	0%, 0	0.881
Leukopenia						
Cediranib plus Olaparib vs. Olaparib	2	0.932	0.633–1.372	0.721	0%, 0	0.369
Neutropenia						
Cediranib plus Olaparib vs. Olaparib	2	1.273	0.809–2.003	0.297	0%, 0	0.475
Bevacizumab plus Olaparib (or Niraparib) vs. Olaparib (or Niraparib)	2	0.744	0.453–1.220	0.241	0%, 0	0.358
Thrombocytopenia						
Cediranib plus Olaparib vs. Olaparib	2	2.051	1.302–3.229	0.002	0%, 0	0.602
Bevacizumab plus Olaparib (or Niraparib) vs. Olaparib (or Niraparib)	2	0.733	0.432–1.243	0.249	0%, 0	0.335
Nausea						
Cediranib plus Olaparib vs. Olaparib	2	1.113	0.987–1.256	0.081	13.1%, 0.0016	0.283
Bevacizumab plus Olaparib (or Niraparib) vs. Olaparib (or Niraparib)	2	0.950	0.613–1.473	0.818	81.6%, 0.0832	0.020
Vomiting						
Cediranib plus Olaparib vs. Olaparib	2	1.305	1.024–1.663	0.031	0%, 0	0.799
Bevacizumab plus Olaparib (or Niraparib) vs. Olaparib (or Niraparib)	2	1.031	0.297–3.580	0.961	89.3%, 0.7240	0.002
Diarrhea						
Cediranib plus Olaparib vs. Olaparib	2	9.912	0.515–190.778	0.129	89.2%, 4.1064	0.002
Bevacizumab plus Olaparib (or Niraparib) vs. Olaparib (or Niraparib)	2	0.605	0.429–0.853	0.004	22.0%, 0.0396	0.257
Abdominal pain						
Cediranib plus Olaparib vs. Olaparib	2	1.414	1.088–1.837	0.010	44.3%, 0.1663	0.180
Constipation						
Cediranib plus Olaparib vs. Olaparib	2	1.618	0.363–7.218	0.529	75.6%, 0.9284	0.043
Proteinuria						
Bevacizumab plus Olaparib (or Niraparib) vs. Olaparib (or Niraparib)	2	33.136	4.711–233.077	<0.001	0%, 0	0.685
Fatigue						
Cediranib plus Olaparib vs. Olaparib	2	1.244	1.110–1.394	<0.001	0%, 0	0.351
Bevacizumab plus Olaparib (or Niraparib) vs. Olaparib (or Niraparib)	2	1.295	1.071–1.566	0.008	0%, 0	0.491
Headache						
Cediranib plus Olaparib vs. Olaparib	2	2.862	1.106–7.409	0.030	70.6%, 0.3500	0.065
Anorexia						
Cediranib plus Olaparib vs. Olaparib	2	1.942	0.946–3.988	0.071	52.9%, 0.1705	0.145
Dyspnea						
Cediranib plus Olaparib vs. Olaparib	2	2.684	0.261–27.651	0.407	66.3%, 2.1025	0.085
Hypertension						
Cediranib plus Olaparib vs. Olaparib	2	14.608	0.951–224.473	0.054	75.4%, 3.1047	0.044
Bevacizumab plus Olaparib (or Niraparib) vs. Olaparib (or Niraparib)	2	5.722	1.037–31.579	0.045	93.4%, 1.4185	<0.001

2 RCTs addressed OS outcome. There was no significant heterogeneity observed across trials (I^2^ = 29.9%, Tau^2^ = 0.0197). The results, derived from a fixed-effects model, indicated that compared with PARP inhibitor or antiangiogenic monotherapy, combination therapy led to an improvement in OS, but with no statistical significance (HR = 0.885, 95% CI = 0.737–1.063) (Table 2; Figure 2B). The constricted inclusion of merely two trials in the pooled analysis precluded the possibility of conducting a subgroup analysis for OS outcome.

### 3.4 Meta-analysis of safety outcomes

#### 3.4.1 Hematologic AEs

**FIGURE 3:**
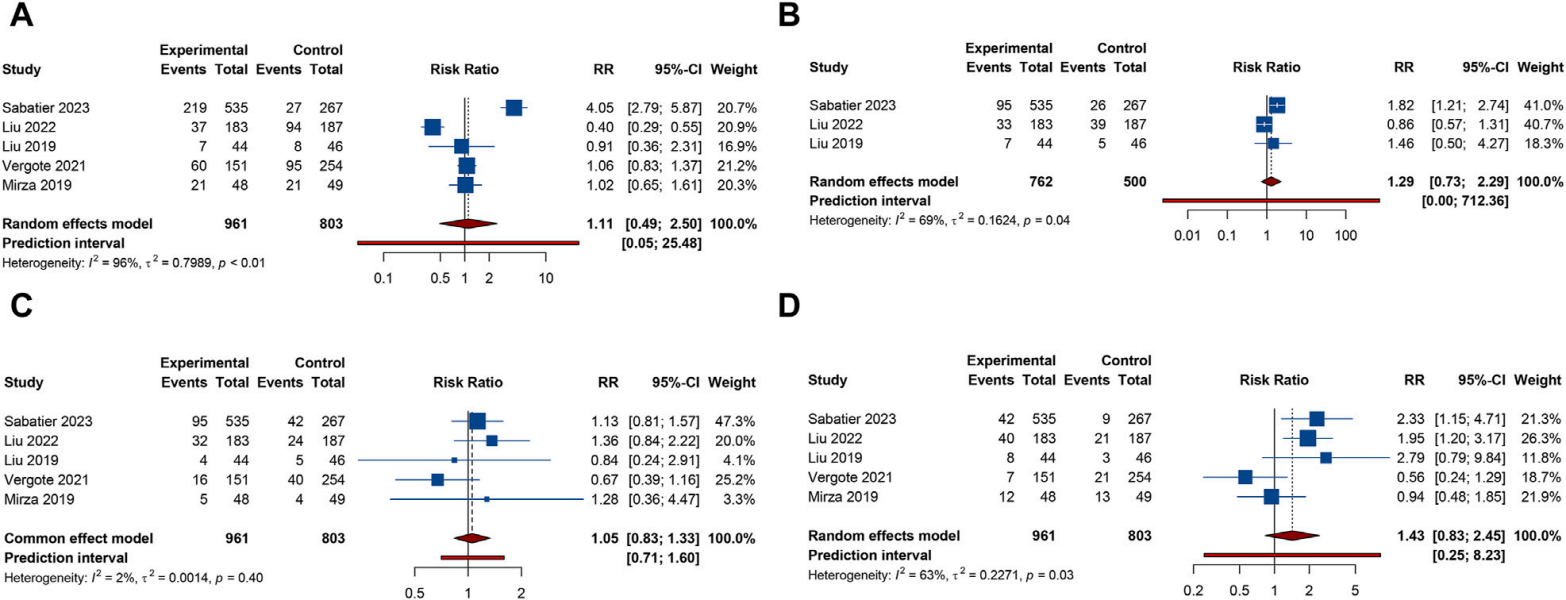
Forest plot of hematologic adverse events after combination therapy with PARP inhibitors and antiangiogenic drugs for ovarian cancer. **(A)** Anemia; **(B)** Leukopenia; **(C)** Neutropenia; **(D)** Thrombocytopenia.

5 studies documented the AEs of anemia, neutropenia, or thrombocytopenia, while leukopenia was examined in 3 trials. The overall analysis proposed that PARP inhibitors plus antiangiogenic agents did not elevate the occurrence of anemia (RR = 1.106, 95% CI = 0.490–2.498; 95% PI = 0.048–25.480; I^2^ = 95.5%, Tau^2^ = 0.7989), leukopenia (RR = 1.293, 95% CI = 0.732–2.285; 95% PI = 0.002–712.363; I^2^ = 68.9%, Tau^2^ = 0.1624), neutropenia (RR = 1.054, 95% CI = 0.833–1.332; 95% PI = 0.706–1.597; I^2^ = 1.6%, Tau^2^ = 0.0014), and thrombocytopenia (RR = 1.427, 95% CI = 0.832–2.449; 95% PI = 0.248–8.227; I^2^ = 62.7%, Tau^2^ = 0.2271) relative to the isolated application of either PARP inhibitors or antiangiogenic medications (Table 2; Figure 3). However, the subgroup analysis indicated that cediranib plus olaparib posed a higher risk for thrombocytopenia (RR = 2.051, 95% CI = 1.302–3.229; I^2^ = 0%, Tau^2^ = 0) compared with olaparib monotherapy (Table 3; Supplementary Figure S2).

#### 3.4.2 Gastrointestinal AEs

**FIGURE 4:**
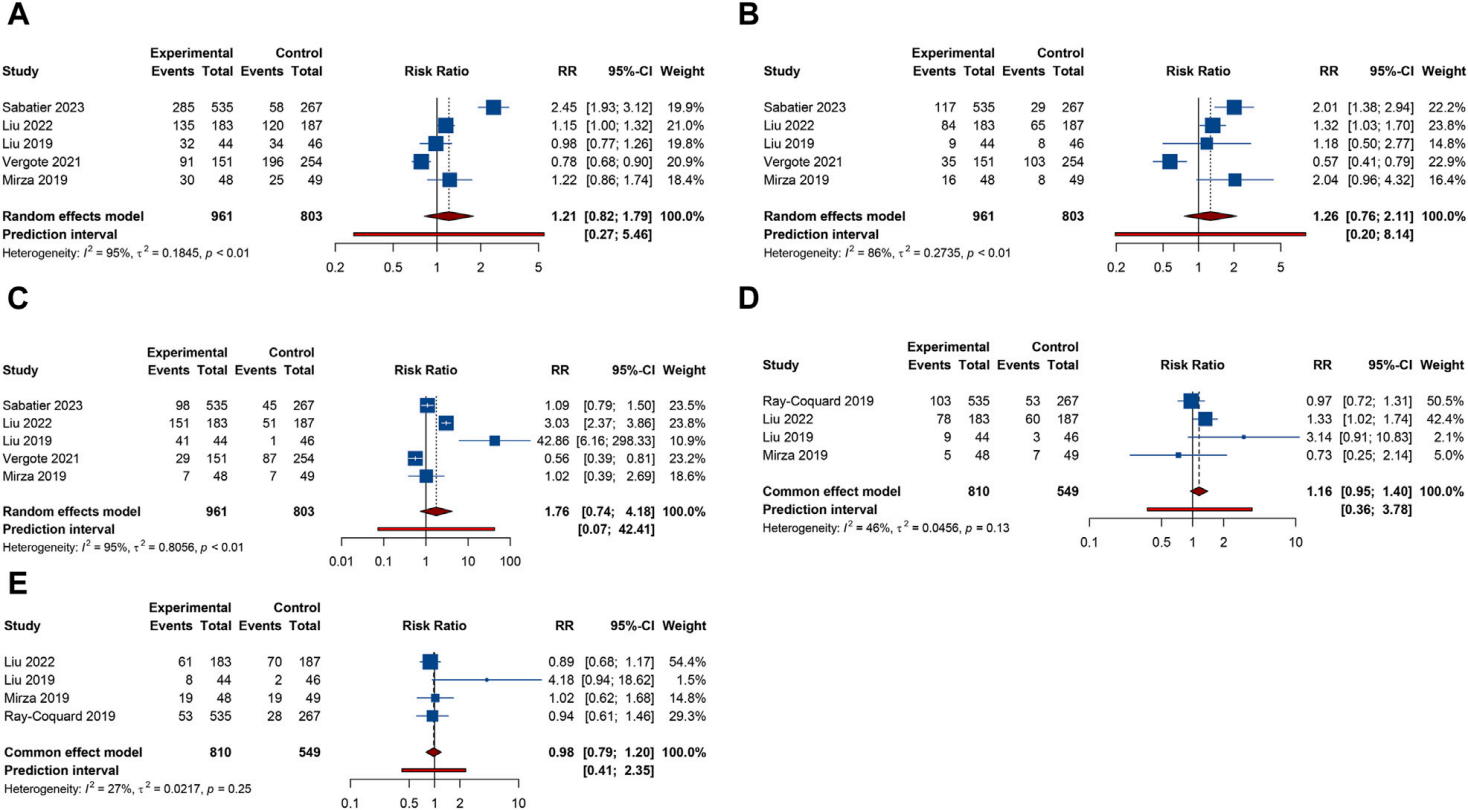
Forest plot of gastrointestinal adverse events after combination therapy with PARP inhibitors and antiangiogenic drugs for ovarian cancer. **(A)** Nausea; **(B)** Vomiting; **(C)** Diarrhea; **(D)** Abdominal pain; **(E)** Constipation.

5 RCTs furnished data on gastrointestinal AEs, including nausea, vomiting, or diarrhea. The overall analysis revealed that compared with PARP inhibitor or antiangiogenic monotherapy, combination therapy with PARP inhibitors and antiangiogenic drugs did not raise the risks of nausea (RR = 1.210, 95% CI = 0.818–1.788; 95% PI = 0.268–5.459; I^2^ = 94.7%, Tau^2^ = 0.1845), vomiting (RR = 1.264, 95% CI = 0.756–2.115; 95% PI = 0.196–8.139; I^2^ = 86.4%, Tau^2^ = 0.2735), and diarrhea (RR = 1.757, 95% CI = 0.739–4.177; 95% PI = 0.073–42.406; I^2^ = 94.7%, Tau^2^ = 0.8056) (Table 2; Figures 4A–C). Subgroup analysis indicated that compared with olaparib monotherapy, combination therapy with cediranib and olaparib escalated vomiting risk (RR = 1.305, 95% CI = 1.024–1.663; I^2^ = 0%, Tau^2^ = 0). Additionally, the combination of bevacizumab and olaparib (or niraparib) was associated with a reduced likelihood of diarrhea relative to the monotherapeutic application of olaparib (or niraparib) (RR = 0.605, 95% CI = 0.429–0.853; I^2^ = 22.0%, Tau^2^ = 0.0396) (Table 3; Supplementary Figure S3).

4 trials provided information on abdominal pain or constipation. The findings from these studies suggested that the combination therapy of PARP inhibitors and antiangiogenic agents was not associated with an increased incidence of abdominal pain (RR = 1.156, 95% CI = 0.952–1.402; 95% PI = 0.364–3.778; I^2^ = 46.2%, Tau^2^ = 0.0456) and constipation (RR = 0.976, 95% CI = 0.791–1.204; 95% PI = 0.410–2.347; I^2^ = 27.3%, Tau^2^ = 0.0217) compared with PARP inhibitor or antiangiogenic drug monotherapy (Table 2; Figures 4D,E). Nonetheless, the combination of bevacizumab and olaparib was linked with a considerable increase in the risk of abdominal pain relative to the use of olaparib alone (RR = 1.414, 95% CI = 1.088–1.837; I^2^ = 44.3%, Tau^2^ = 0.1663) (Table 3; Supplementary Figure S3).

#### 3.4.3 Renal and urinary AEs

**FIGURE 5:**
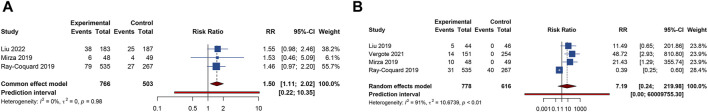
Forest plot of renal and urinary adverse events after combination therapy with PARP inhibitors and antiangiogenic drugs for ovarian cancer. **(A)** Urinary tract infection; **(B)** Proteinuria.

Urinary tract infection was reported in 3 RCTs. Patients receiving combination therapy with PARP inhibitors and antiangiogenic drugs exhibited a statistically significant increase in the incidence of urinary tract infection compared with monotherapy (RR = 1.500, 95% CI = 1.114–2.021; 95% PI = 0.218–10.346; I^2^ = 0%, Tau^2^ = 0) (Table 2; Figure 5A). Subgroup analysis based on the specific drugs of PARP inhibitors and antiangiogenic therapy was not available for urinary tract infection. Proteinuria outcome was examined in 4 RCTs. The overall analysis indicated that PARP inhibitors plus antiangiogenic agents did not escalate the occurrence of proteinuria relative to monotherapy (RR = 7.195, 95% CI = 0.235–219.980; I^2^ = 90.7%, Tau^2^ = 10.6739) (Table 2; Figure 5B). Yet, subgroup analysis demonstrated that compared with olaparib (or niraparib) monotherapy, bevacizumab plus olaparib (or niraparib) therapy significantly heightened proteinuria risk (RR = 33.136, 95% CI = 4.711–233.077; I^2^ = 0%, Tau^2^ = 0) (Table 3; Supplementary Figure S4).

#### 3.4.4 Other AEs

**FIGURE 6:**
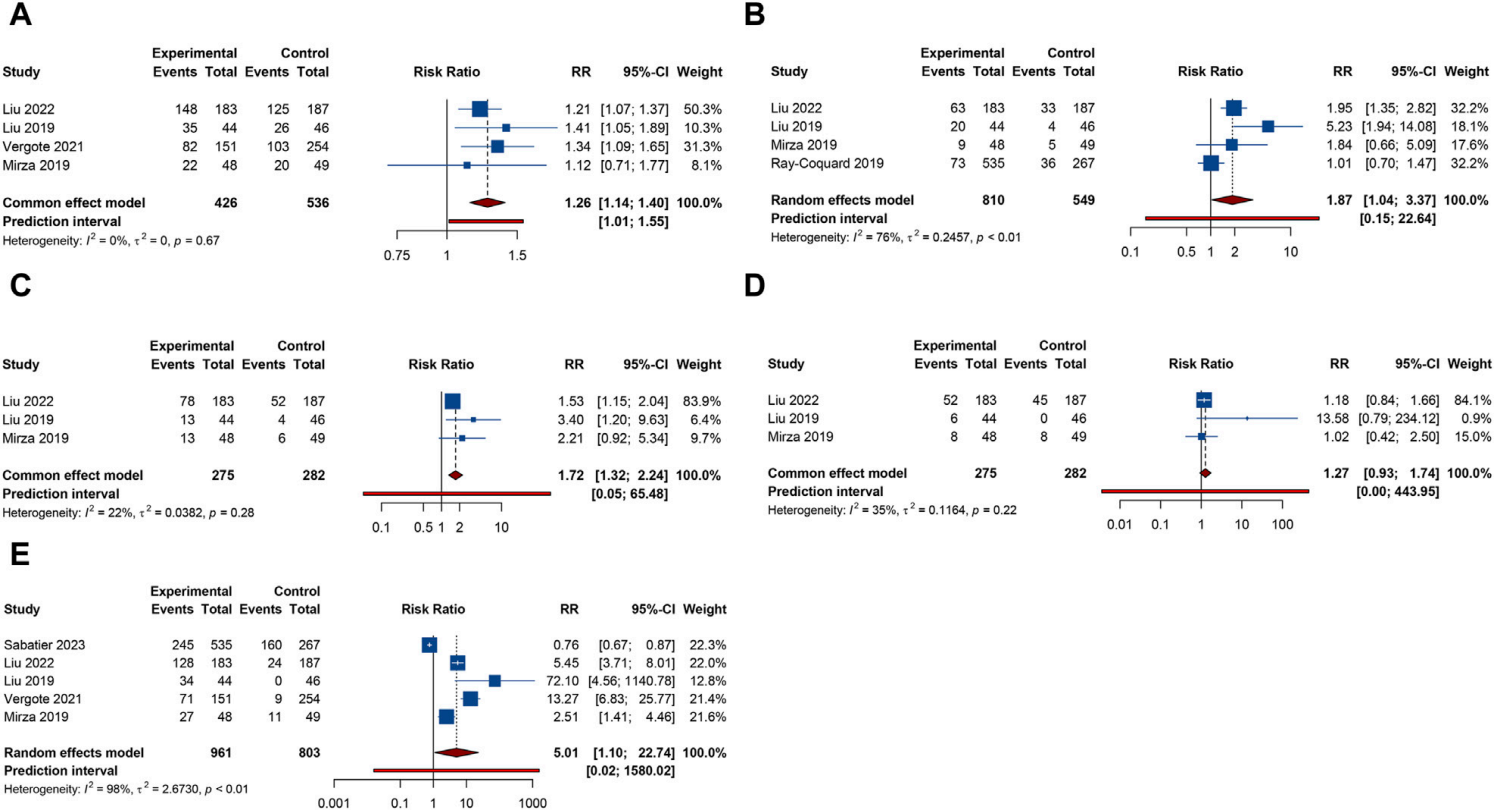
Forest plot of other adverse events after combination therapy with PARP inhibitors and antiangiogenic drugs for ovarian cancer. **(A)** Fatigue; **(B)** Headache; **(C)** Anorexia; **(D)** Dyspnea; **(E)** Hypertension.

4 trials analyzed fatigue or headache. The overall analysis suggested that combination treatment of PARP inhibitors and antiangiogenic drugs significantly increased the risks of fatigue (RR = 1.264, 95% CI = 1.141–1.400; 95% PI = 1.012–1.552; I^2^ = 0%, Tau^2^ = 0) and headache (RR = 1.868, 95% CI = 1.036–3.369; 95% PI = 0.154–22.642; I^2^ = 76.0%, Tau^2^ = 0.2457) (Table 2; Figures 6A,B). Subgroup analysis showed that combination therapy with cediranib and olaparib was related to an increased risk of fatigue (RR = 1.244, 95% CI = 1.110–1.394; I^2^ = 0%, Tau^2^ = 0) and headache (RR = 2.862, 95% CI = 1.106–7.409; I^2^ = 70.6%, Tau^2^ = 0.3500) compared to olaparib monotherapy. Similarly, bevacizumab plus olaparib (or niraparib) therapy was found to heighten fatigue risk compared to olaparib (or niraparib) monotherapy (RR = 1.295, 95% CI = 1.071–1.566; I^2^ = 0%, Tau^2^ = 0) (Table 3; Supplementary Figure S5).

3 RCTs investigated anorexia or dyspnea. The incidence of anorexia was notably higher in patients receiving combined PARP inhibitor and antiangiogenic therapy than in those on either treatment alone (RR = 1.718, 95% CI = 1.320–2.235; 95% PI = 0.050–65.480; I^2^ = 22.3%, Tau^2^ = 0.0382). However, this combination did not correlate with a higher rate of dyspnea (RR = 1.272, 95% CI = 0.928–1.742; 95% PI = 0.004–443.951; I^2^ = 34.8%, Tau^2^ = 0.1164) (Table 2; Figures 6C, D). Further examination of subgroups did not reveal a significant link between cediranib plus olaparib therapy and the onset of anorexia and dyspnea (all *p* > 0.05) (Table 3; Supplementary Figure S5).

5 RCTs focused on hypertension outcome. The combined therapy of PARP inhibitors and antiangiogenic agents was found to escalate hypertension risk (RR = 5.009, 95% CI = 1.103–22.744; 95% PI = 0.016–1580.021; I^2^ = 98.2%, Tau^2^ = 2.6730) (Table 2; Figure 6E). Subgroup analysis demonstrated that the combined treatment with bevacizumab and either olaparib or niraparib led to a significant increase in hypertension incidence relative to olaparib or niraparib monotherapy (RR = 5.722, 95% CI = 1.037–31.579; I^2^ = 93.4%, Tau^2^ = 1.4185) (Table 3; Supplementary Figure S5).

### 3.5 Sensitivity analysis and publication bias

Given the limited number of studies incorporated into the pooled analyses, which might impact the robustness of sensitivity analysis and the evaluation of publication bias, we only carried out these assessments for PFS, the outcome with the largest number of studies included. To ensure the reliability of our findings, we employed the leave-one-out method for the sensitivity analysis. This approach confirmed the stability of the pooled PFS result (Supplementary Figure S6). Begg’s and Egger’s tests were applied to evaluate publication bias. The results indicated no significant publication bias in PFS outcome (Begg’s test: *p* = 0.452, Egger’s test: *p* = 0.420). The funnel plots were presented in Supplementary Figure S7.

### 3.6 Trial sequential analysis results

**FIGURE 7:**
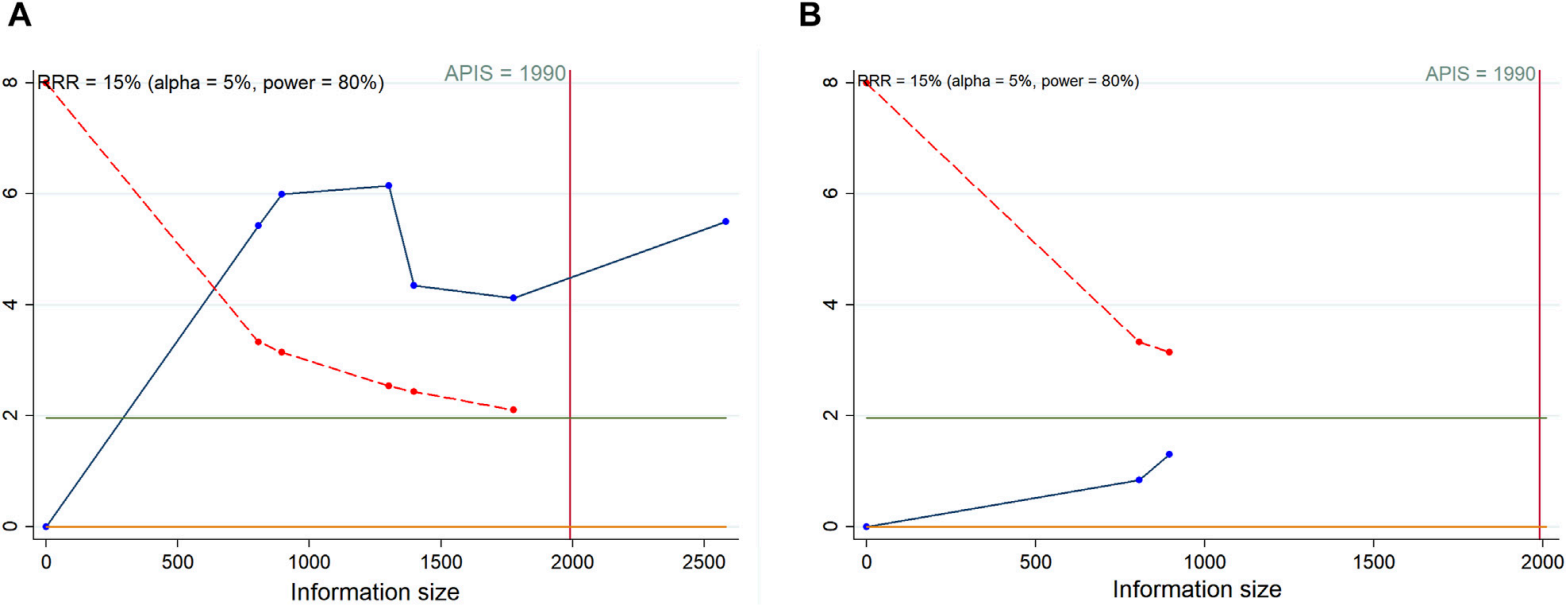
Trial sequential analysis of progression-free survival **(A)** and overall survival **(B)** after combination therapy with PARP inhibitors and antiangiogenic drugs for ovarian cancer. Red inward-sloping line to the left represents trial sequential monitoring boundary. Blue line represents evolution of cumulative Z-score. Horizontal green lines represent the conventional boundaries for statistical significance. Heterogeneity-adjusted required information size to demonstrate or reject 15% relative risk (*a priori* estimate) of mortality risk (with alpha of 5% and beta of 20%) is 1990 patients for PFS and OS (vertical red line). Cumulative Z-curve crossing the trial sequential monitoring boundary or the APIS boundary provides firm evidence of effect.

As shown in Figure 7, we calculated a RIS of 1990 for PFS and OS. The cumulative Z-curve for PFS traversed both the RIS boundary and the trial sequential monitoring boundary, implying a relatively definitive result for PFS. Conversely, the cumulative Z-curve for OS failed to cross either boundary, suggesting that a solid conclusion regarding OS cannot be drawn due to potential false positive. Regarding AEs, definitive conclusions can be inferred for urinary tract infection, fatigue, and anorexia, as only their cumulative Z-curves managed to cross the trial sequential monitoring boundary or RIS boundary (Supplementary Figures S8–S11).

## 4 Discussion

PARP inhibitors and antiangiogenic agents, both demonstrating promising efficacy as standalone treatments, have garnered particular attention to their combination due to minimal overlapping toxicities (Mirza et al., 2016; Coleman et al., 2017; Moore et al., 2018; González-Martín et al., 2019). The groundbreaking PAOLA-1/ENGOT-ov25 trial, which released its findings in 2019, included 806 patients who were divided in a 2:1 ratio to either receive a combination of bevacizumab and olaparib or placebo as the first-line maintenance treatment following response to a regimen of chemotherapy and bevacizumab. The addition of maintenance olaparib yielded a significant benefit in terms of PFS (HR = 0.59, 95% CI = 0.49–0.72) (Ray-Coquard et al., 2019). However, a subsequent joint analysis of the SOLO1 and PAOLA-1/ENGOT-ov25 trials indicated that the addition of bevacizumab to olaparib did not appear to enhance PFS compared with olaparib alone (HR = 0.71, 95% CI = 0.45–1.09) (Vergote et al., 2021). Despite previous network meta-analysis reporting significant benefit of PARP inhibitor and angiogenesis inhibitor monotherapy in improving PFS compared to placebo (Feng et al., 2019), there is currently still a lack of meta-analysis directly comparing the efficacy and safety of combined therapy with these two drugs *versus* monotherapy for patients with OC. Therefore, we performed a systematic review and meta-analysis of previous RCTs, and the pooled results demonstrated that combination therapy with PARP inhibitors and antiangiogenic drugs significantly improved PFS, but also increased the risks of AEs such as urinary tract infection, fatigue, headache, anorexia, and hypertension compared with monotherapy with either a PARP inhibitor or an antiangiogenic agent. Given the immature OS outcome in several trials, this meta-analysis obtained OS data from only two RCTs, and the combined results did not confirm the OS benefits of combination therapy compared to monotherapy.

Experimental studies have indicated pathways through which the joint administration of PARP inhibitors and antiangiogenic treatments could enhance outcomes in OC (Lim et al., 2014; Ivy et al., 2016). The study suggested a synergistic effect, with direct and indirect modulation of the tumor cell genome-chiefly through alterations in the tumor microenvironment-potentially underpinning the improved therapeutic efficacy (Ivy et al., 2016). One such mechanism involves the hypoxic conditions induced by antiangiogenic agents (Ueda et al., 2017), which have been observed to attenuate the expression and functionality of the homologous recombination protein RAD51 in neoplastic cells (Chan et al., 2010b). This downregulation of RAD51 under hypoxic conditions was further validated *in vivo* through immunofluorescent imaging of mouse model tumors (Bindra et al., 2004). Additionally, the suppression of VEGFR3 in OC cells has been correlated with reduced levels of the tumor suppressor proteins BRCA1 and BRCA2 (Lim et al., 2014). On the flip side, PARP1-deficient mice exhibited impaired angiogenic responses to growth factors (Tentori et al., 2007). Preclinical models also revealed that high levels of PARP1 expression enhance angiogenesis in epithelial OC by modulating VEGF-A (Wei et al., 2016). The silencing of PARP1 in SKOV3 cells markedly lowered VEGF-A mRNA and protein levels, thus supporting the rationale for the combination of both agents (Le Saux et al., 2021). Nonetheless, the precise biological underpinnings of these therapeutic combinations remain elusive, potentially differ with each antiangiogenic agents, and have yet to be confirmed in clinical settings. Further research is needed to precisely delineate the mechanisms by which this combination exerts its antineoplastic effects. Beyond demonstrating PFS benefit from combination therapy of PARP inhibitors and antiangiogenic agents, our further subgroup analysis validated that combination therapy with olaparib and bevacizumab improved PFS compared with bevacizumab monotherapy. The PAOLA-1 study, a randomized, double-blind, phase III trial, compared the efficacy of olaparib-bevacizumab combined treatment against bevacizumab-placebo in OC patients. The PFS outcome from this study lent credence to the proposition that olaparib, when added to bevacizumab as an initial maintenance therapy, could offer substantial clinical benefit. These findings have led to the authorization by the US Food and Drug Administration (FDA) and European Medicines Agency (EMA) of the olaparib-bevacizumab combination for maintenance therapy in the OC patients (Ray-Coquard et al., 2019; Salutari et al., 2024). Updated analysis from the PAOLA-1/ENGOT-ov25 trial further corroborated that olaparib plus bevacizumab combination therapy significantly prolonged PFS compared with bevacizumab plus placebo treatment (HR = 0.63, 95% CI = 0.53–0.74) (Ray-Coquard et al., 2023).

Our study did not substantiate an OS benefit of combination therapy with PARP inhibitors and antiangiogenic drugs in OC patients. While several trials have included OS as an exploratory endpoint, conclusive results on OS have not been realized owing to the insufficient follow-up time up to the data cutoff point (Mirza et al., 2019; Liu et al., 2022). Additionally, RCTs analyzing the outcome of OS reported no significant effect regarding the combined treatment of PARP inhibitors and antiangiogenic agents for OS (Liu et al., 2019; Ray-Coquard et al., 2023). A more recent analysis from a phase II randomized, open-label trial compared the median OS of patients treated with the cediranib-olaparib combination (44.2 months) against those receiving olaparib as a single agent (33.3 months). The HR for this comparison stood at 0.64 with a 95% CI ranging from 0.36 to 1.11, indicating no substantial improvement (Liu et al., 2019). Comprehensive OS results from the PAOLA-1/ENGOT-ov25 trial suggested a slight, non-significant trend towards better OS for patients treated with the combination of olaparib and bevacizumab compared to those receiving bevacizumab with placebo (HR = 0.92, 95% CI = 0.76–1.12) (Ray-Coquard et al., 2023). Besides significant disparities in follow-up duration, the included two RCTs also exhibited considerable differences in the number of patients included in the combination therapy and monotherapy groups. Such variations could potentially influence the pooled results for OS to a certain extent. Consequently, the conclusions drawn from this meta-analysis on the impact of combination therapy on OS in OC patients will require updates in light of forthcoming results from mature OS outcome.

Numerous phase II/III randomized trials have highlighted the therapeutic gains of combining PARP inhibitors with antiangiogenic agents (Liu et al., 2014; Liu et al., 2020; Lorusso et al., 2020; Mirza et al., 2020; Hardesty et al., 2021), yet the elevated risk of AEs warrants attention. The safety profiles for such combined therapies align broadly with those observed for each treatment in isolation, with common all-grade AEs including fatigue, diarrhea, hypertension, and nausea (Alvarez Secord et al., 2021). Our study demonstrated that OC patients receiving combination therapy of PARP inhibitors and antiangiogenic agents experienced a higher occurrence of urinary tract infection, fatigue, headache, anorexia, and hypertension than those on PARP inhibitor or antiangiogenic agent monotherapy. AEs were typically controlled with supportive care and dosage modifications, rarely necessitating cessation of therapy (Pujade-Lauraine et al., 2017; Moore et al., 2018; Ray-Coquard et al., 2019). Notably, myelosuppression stands out as a significant clinical concern with PARP inhibitor combinations due to its potential severity and life-threatening nature, with hematological toxicities being predominant (Ren et al., 2021). Further analysis within our study revealed an increased risk of thrombocytopenia with the cediranib-olaparib combination compared to olaparib alone, underscoring the necessity for thorough blood evaluations and vigilant monitoring for blood-related toxicities in patients undergoing this treatment. In addition, our subgroup analysis indicated that the combination of cediranib and olaparib increased the incidence of vomiting, abdominal pain, fatigue, and headache compared with olaparib monotherapy. Similarly, bevacizumab combined with olaparib (or niraparib) increased the risk of proteinuria, fatigue, and hypertension compared with olaparib (or niraparib) monotherapy. Cediranib and bevacizumab exhibit distinct safety profiles reflective of their differing mechanisms of action, with the most common AEs for cediranib being fatigue and vomiting (Ledermann et al., 2016), while hypertension is frequently reported with bevacizumab maintenance (Burger et al., 2011; Perren et al., 2011). Proteinuria also merits attention as an AE of interest in bevacizumab treatment (Alvarez Secord et al., 2021). Patients on either cediranib or bevacizumab often require management strategies for hypertension, including antihypertensive medications, and should have their blood pressure closely monitored (Ivy et al., 2016). Intriguingly, our subgroup analysis also revealed that the combination therapy of bevacizumab and olaparib (or niraparib) was associated with a lower incidence of diarrhea, suggesting differential pathways of AE manifestation whose mechanisms remain to be elucidated. Our findings accentuate the necessity for clinicians to be vigilant of AEs such as thrombocytopenia, vomiting, abdominal pain, urinary tract infection, proteinuria, fatigue, headache, anorexia, and hypertension when administering combinatorial PARP inhibitors and antiangiogenic therapy in clinical practice. It is also critical to acknowledge the heightened costs linked to combination treatments, which stem not only from the drugs themselves but also from the necessary healthcare services to administer the treatment and manage any associated toxicities (Hockings and Miller, 2023).

However, AEs that have not been statistically confirmed in our study should not be overlooked, as the wide 95% CIs for the RRs suggests instability in the results (such as diarrhea, proteinuria, constipation, etc.). Therefore, in addition to the various AEs confirmed by this study, it is still necessary in clinical practice to promptly observe and identify any AEs caused by the combination therapy of PARP inhibitors and antiangiogenic agents, and to take timely measures for treatment and control.

There are still several undeniable limitations in present research. First, despite an exhaustive search strategy, the number of studies incorporated into our analysis remains limited. This paucity is likely due to the formidable difficulties encountered in enlisting individuals with OC. Second, the heterogeneity observed across the studies in terms of PFS and majority of AEs may be attributed to variable confounding factors, including disease setting, treatment line, the stage of disease, follow-up duration, therapy modality, treatment duration, drug dosage and diverse ethnic backgrounds of the participants treated with PARP inhibitors and antiangiogenic agents. These confounding factors may also exert an impact on the combined efficacy and safety results. Third, the outcomes of TSA indicated a need for a broader sample size to lend credence to the conclusions drawn regarding OS and the majority of AEs. Furthermore, the limited number of participants in the monotherapy group may lead to instability in the final results, resulting in a wide 95% CI. This issue could be addressed by increasing the sample size. Fourth, the constrained volume of studies that met the inclusion criteria restricts a more nuanced assessment of how combination therapies influence PFS, OS, and AEs across various OC subtypes, such as those delineated by BRCA mutation or homologous recombination deficiency (HRD) status.

## 5 Conclusion

Through a meta-analysis of RCTs, our research demonstrated that combination therapy with PARP inhibitors and antiangiogenic agents significantly improved PFS compared with PARP inhibitor or antiangiogenic agent monotherapy. However, the present pooled analysis failed to substantiate an OS benefit of combination treatment, since the original trial data concerning OS were immature. Moreover, the combination of PARP inhibitors and antiangiogenic drugs increased the risks of AEs, including thrombocytopenia, vomiting, abdominal pain, urinary tract infection, proteinuria, fatigue, headache, anorexia, and hypertension.

## Data Availability

The original contributions presented in the study are included in the article/Supplementary Material, further inquiries can be directed to the corresponding authors.
