# Supplementary material for: Efficacy and safety of PARP inhibitors combined with antiangiogenic agents in the maintenance treatment of ovarian cancer: a systematic review and meta-analysis with trial sequential analysis of randomized controlled trials
Source: Front Pharmacol. 2024 Mar 22;15:1372077. doi: 10.3389/fphar.2024.1372077 (PMC10995238; doi:10.3389/fphar.2024.1372077)
Supplement: Supplementary file 1 [file Table1.docx]

| TABLE S1 Quality analysis of the included studies by modified Jadad scale. | | | | | | |
| --- | --- | --- | --- | --- | --- | --- |
| Study | Randomization | Randomization concealment | Double blind | Withdrawals and dropouts | Score | Study quality |
| Sabatier (2023) | 2 | 2 | 2 | 1 | 7 | High |
| Ray-Coquard (2023) | 2 | 2 | 2 | 1 | 7 | High |
| Liu (2022) | 2 | 2 | 0 | 1 | 5 | High |
| Liu (2019) | 2 | 2 | 0 | 1 | 5 | High |
| Vergote (2021) | 2 | 2 | 0 | 1 | 5 | High |
| Mirza (2019) | 2 | 2 | 0 | 1 | 5 | High |
| Ray-Coquard (2019) | 2 | 2 | 2 | 1 | 7 | High |
