# Supplementary material for: Efficacy and safety of PARP inhibitors combined with antiangiogenic agents in the maintenance treatment of ovarian cancer: a systematic review and meta-analysis with trial sequential analysis of randomized controlled trials
Source: Front Pharmacol. 2024 Mar 22;15:1372077. doi: 10.3389/fphar.2024.1372077 (PMC10995238; doi:10.3389/fphar.2024.1372077)

**FIGURE S1** Subgroup analysis of progression-free survival after combination therapy with PARP inhibitors and antiangiogenic drugs for ovarian cancer.


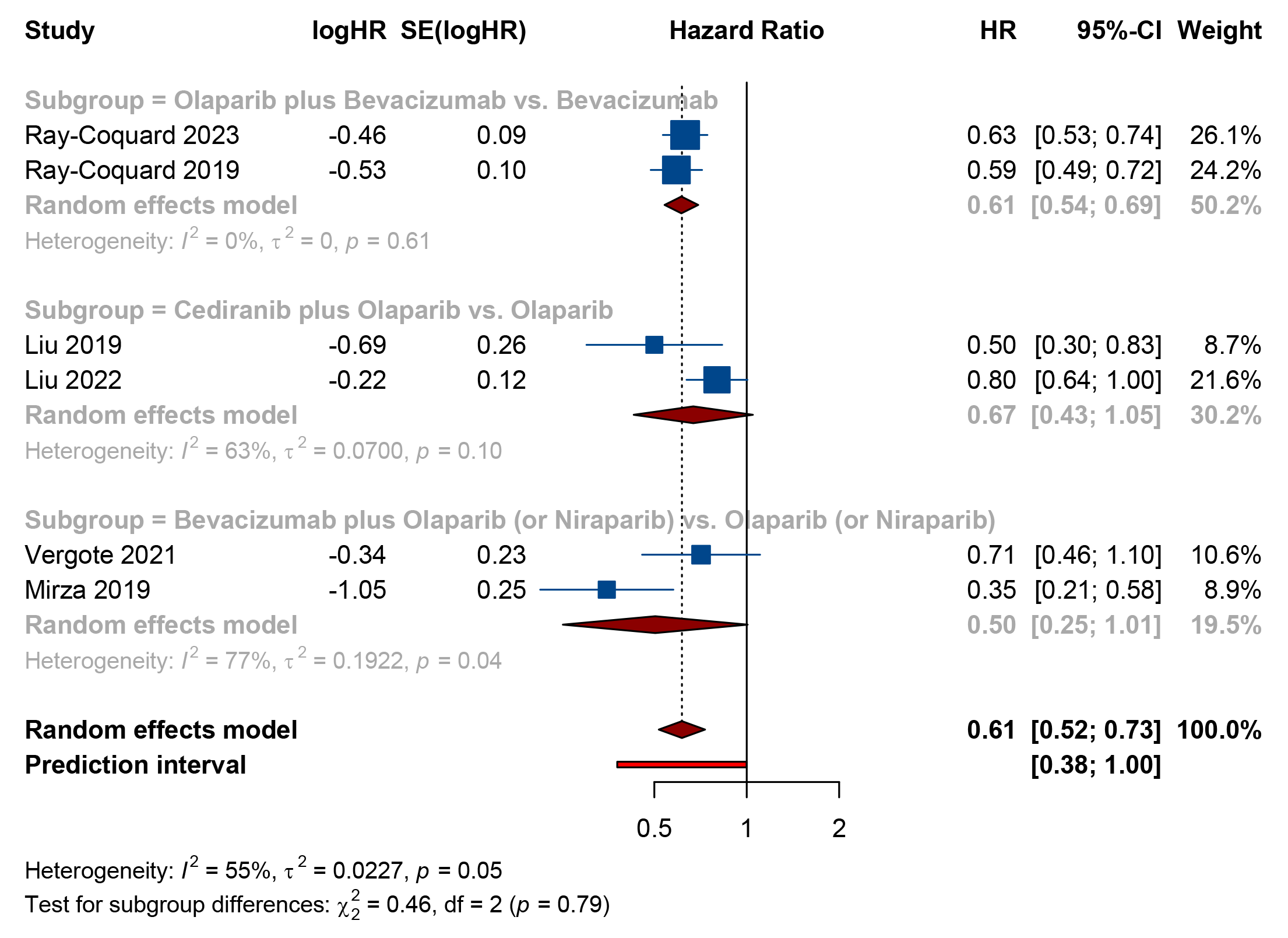


**FIGURE S2** Subgroup analysis of hematologic adverse events after combination therapy with PARP inhibitors and antiangiogenic drugs for ovarian cancer. (A) Anemia; (B) Leukopenia; (C) Neutropenia; (D) Thrombocytopenia.


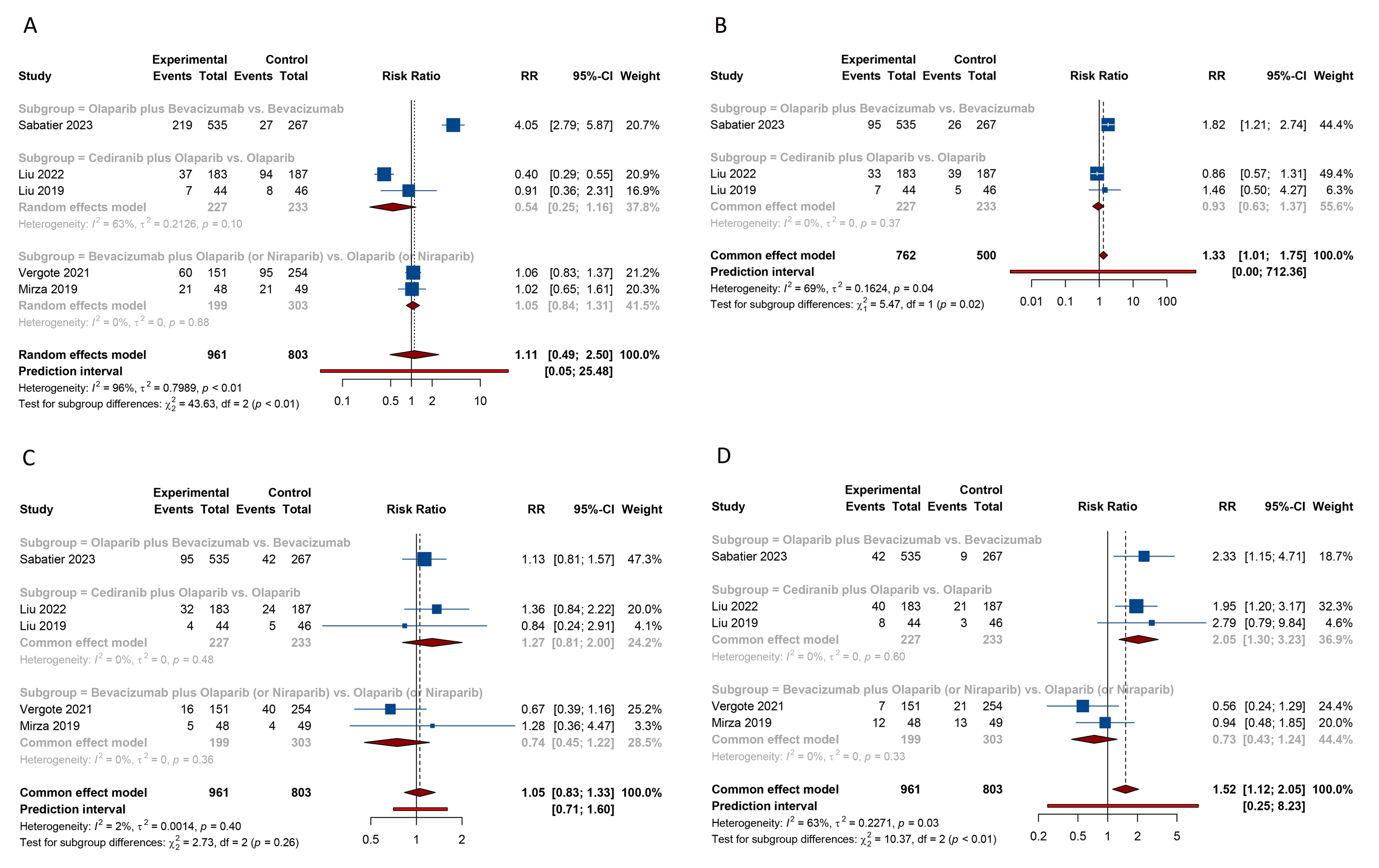


**FIGURE S3** Subgroup analysis of gastrointestinal adverse events after combination therapy with PARP inhibitors and antiangiogenic drugs for ovarian cancer. (A) Nausea; (B) Vomiting; (C) Diarrhea; (D) Abdominal pain; (E) Constipation.


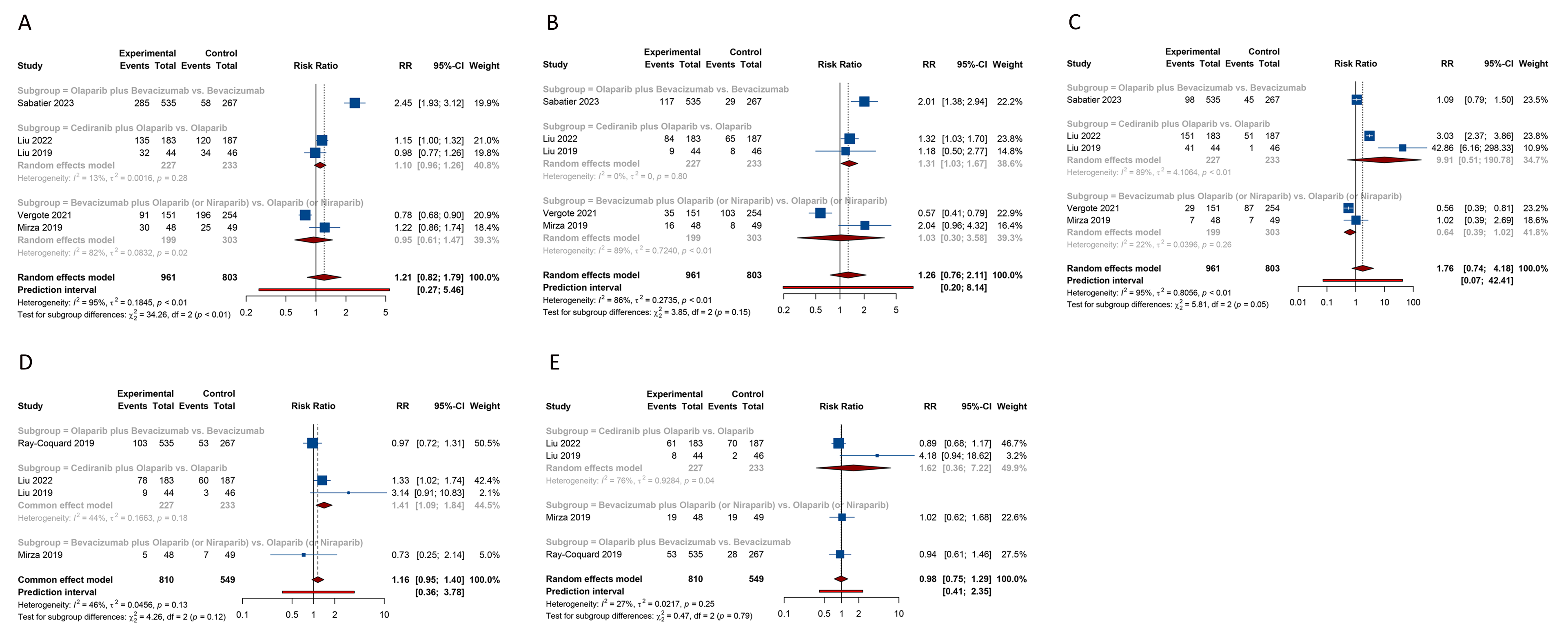


**FIGURE S4** Subgroup analysis of the adverse event of proteinuria after combination therapy with PARP inhibitors and antiangiogenic drugs for ovarian cancer.


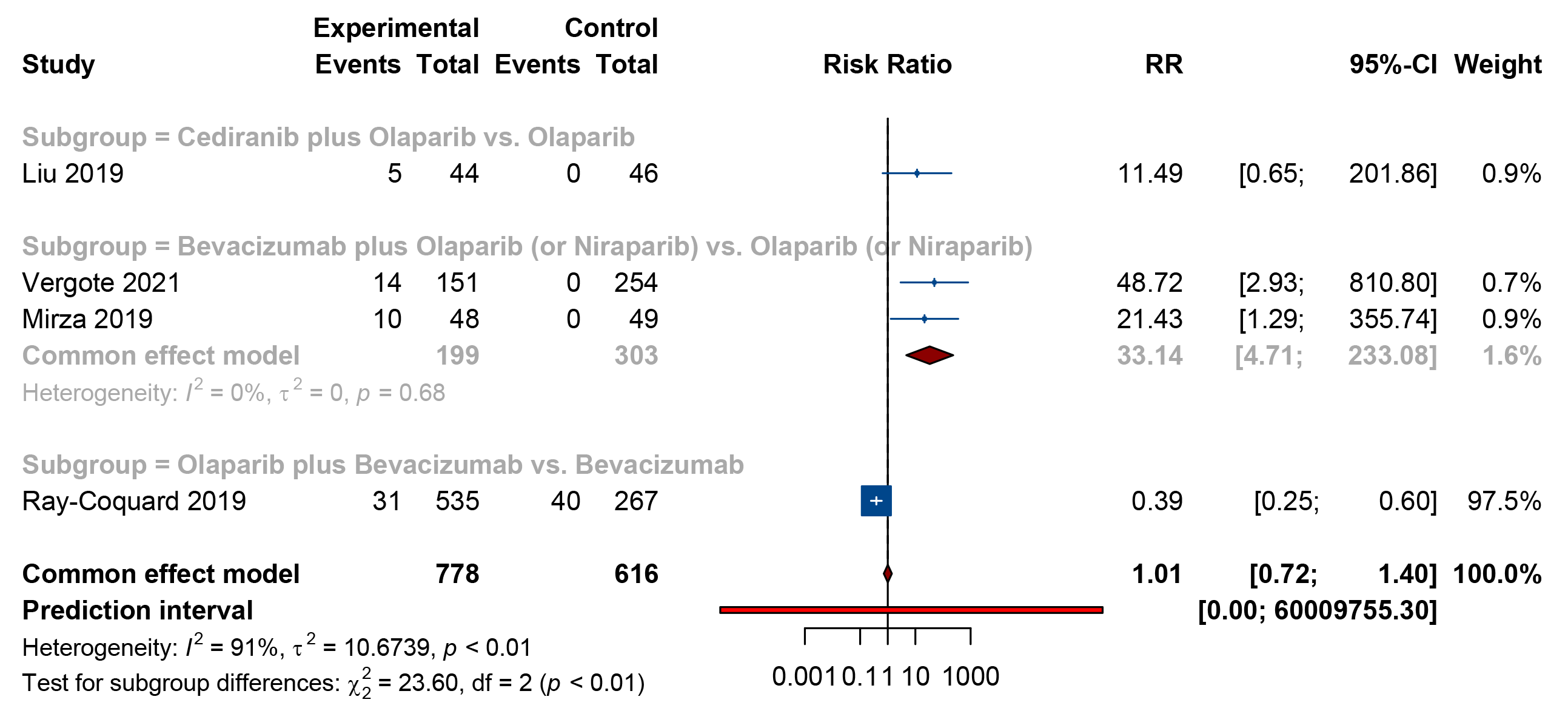


**FIGURE S5** Subgroup analysis of other adverse events after combination therapy with PARP inhibitors and antiangiogenic drugs for ovarian cancer. (A) Fatigue; (B) Headache; (C) Anorexia; (D) Dyspnea; (E) Hypertension.


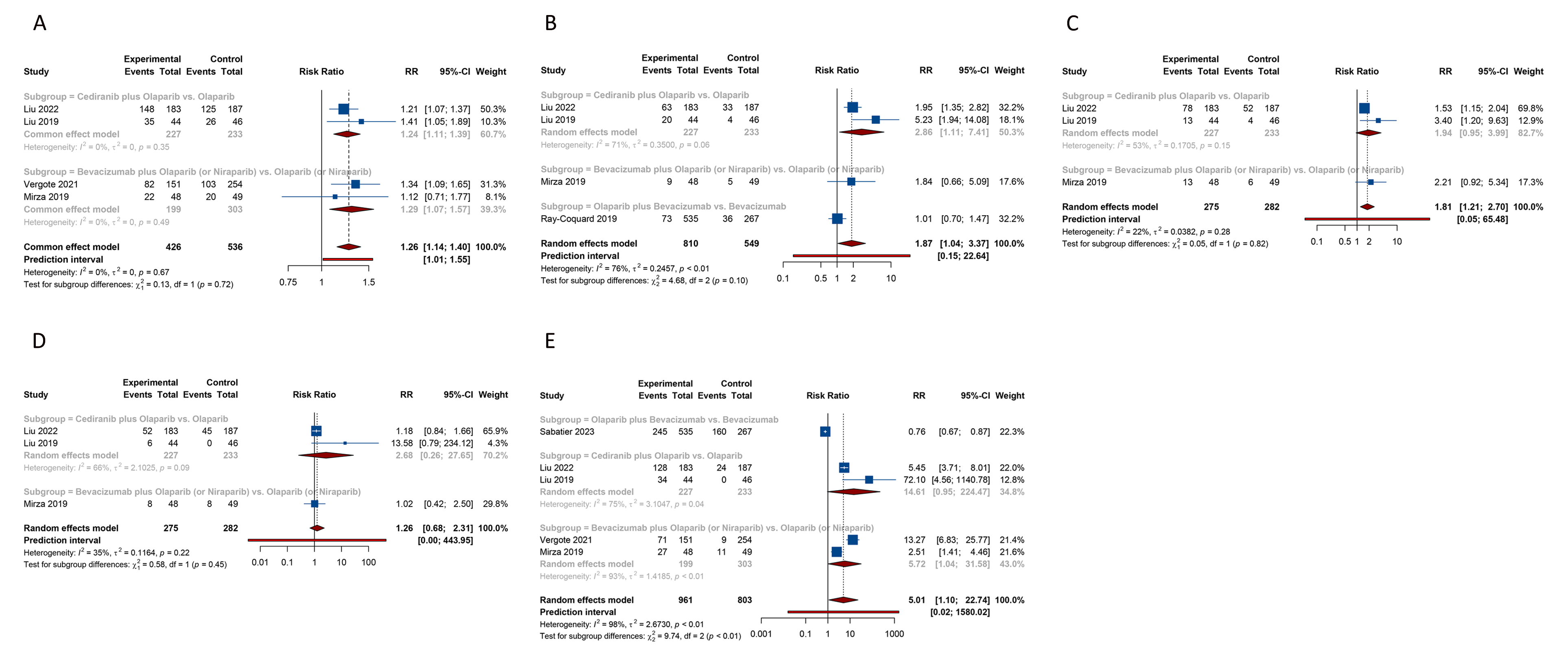


**FIGURE S6** Sensitivity analysis of progression-free survival after combination therapy with PARP inhibitors and antiangiogenic drugs for ovarian cancer.


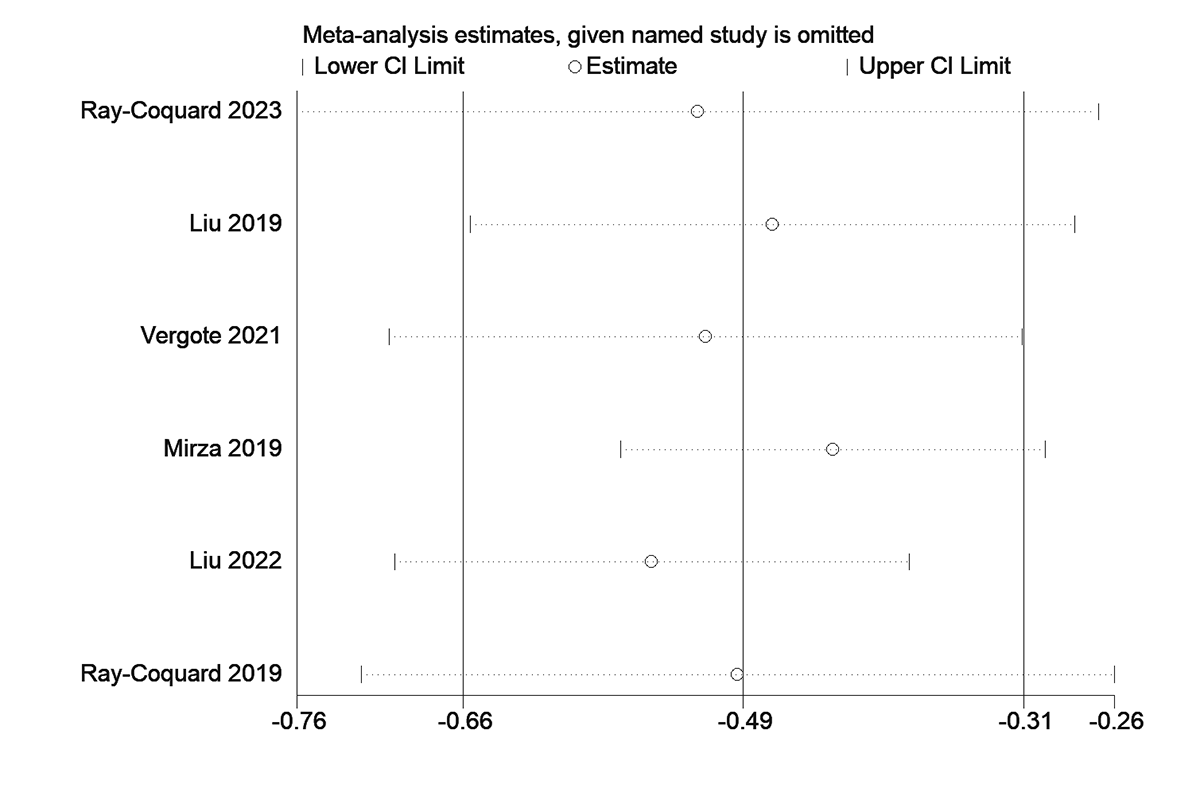


**FIGURE S7** Funnel plot of progression-free survival after combination therapy with PARP inhibitors and antiangiogenic drugs for ovarian cancer.


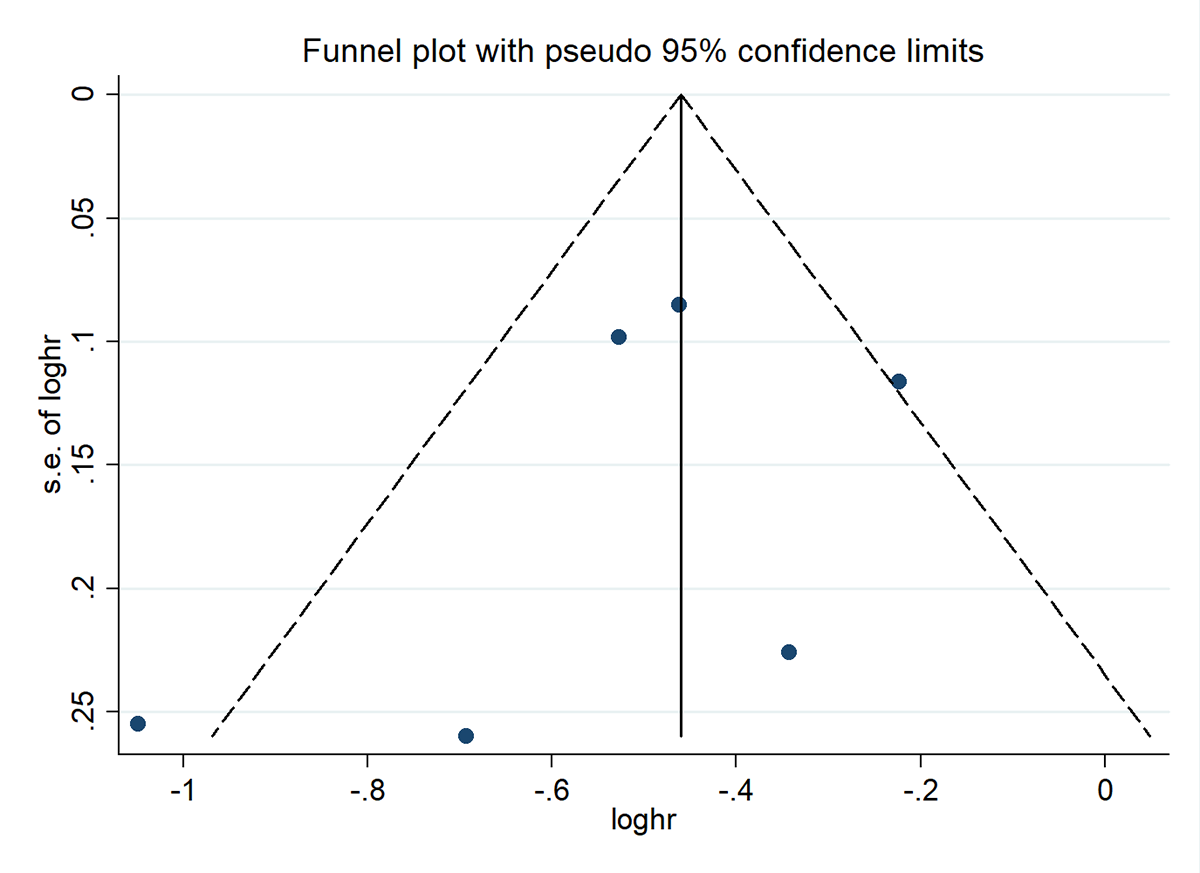


**FIGURE S8** Trial sequential analysis of hematologic adverse events after combination therapy with PARP inhibitors and antiangiogenic drugs for ovarian cancer. (A) Anemia; (B) Leukopenia; (C) Neutropenia; (D) Thrombocytopenia. Uppermost and lowermost red curves represent trial sequential monitoring boundary lines for benefit and harm, respectively. Horizontal green lines represent the conventional boundaries for statistical significance. Inner red lines represent the futility boundary.


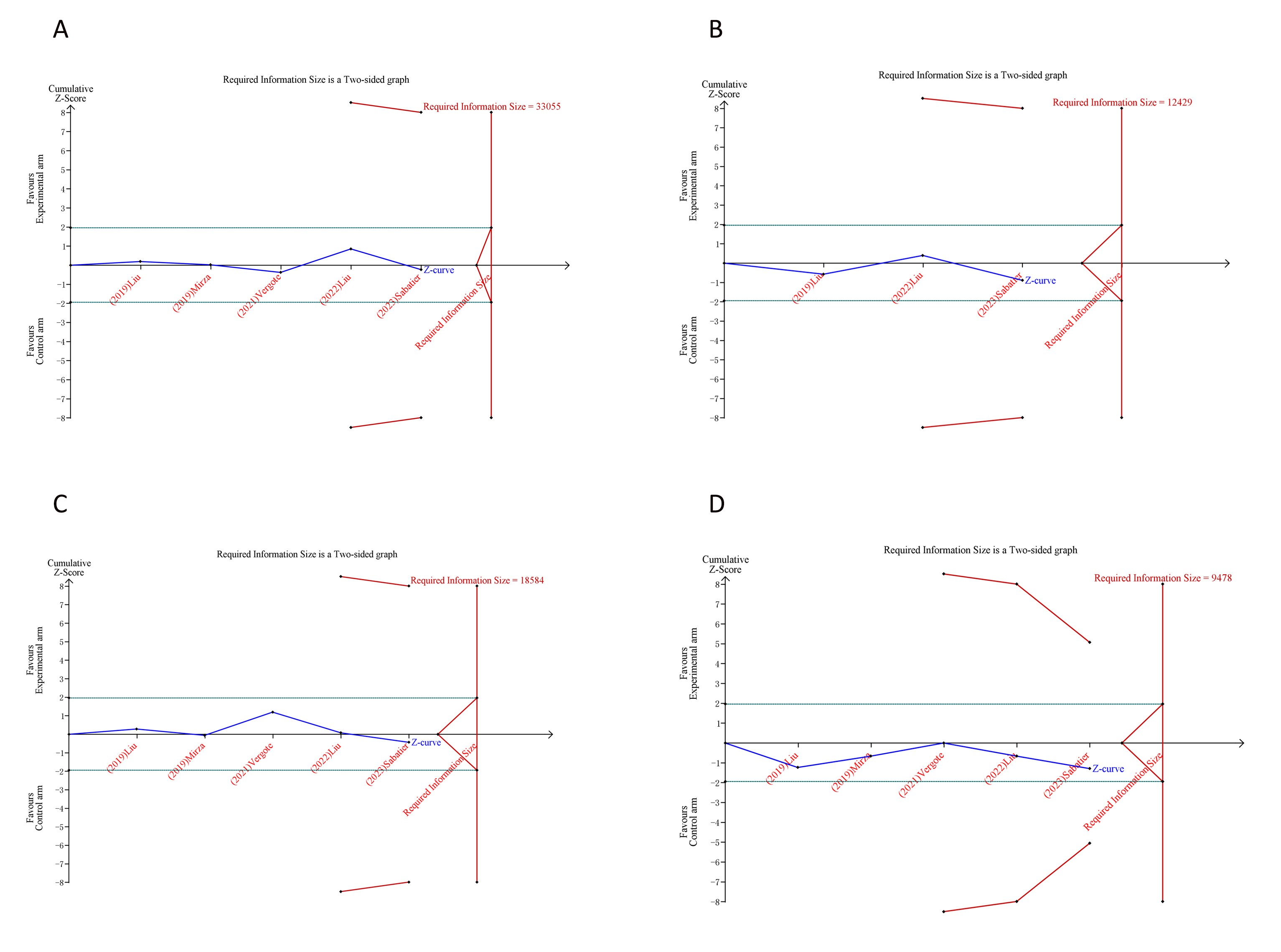


**FIGURE S9** Trial sequential analysis of gastrointestinal adverse events after combination therapy with PARP inhibitors and antiangiogenic drugs for ovarian cancer. (A) Nausea; (B) Vomiting; (C) Diarrhea; (D) Abdominal pain; (E) Constipation. Uppermost and lowermost red curves represent trial sequential monitoring boundary lines for benefit and harm, respectively. Horizontal green lines represent the conventional boundaries for statistical significance. Inner red lines represent the futility boundary.


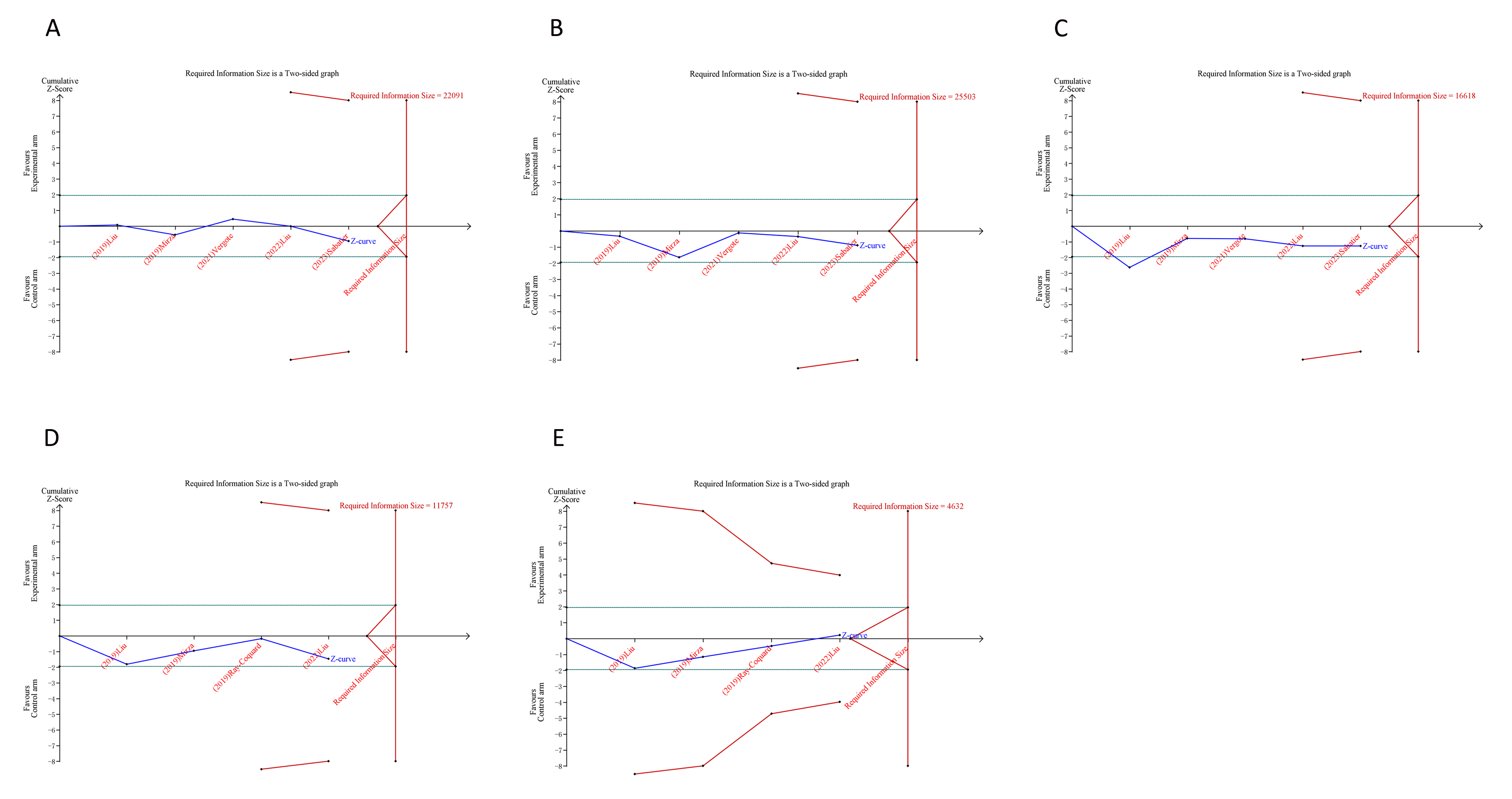


**FIGURE S10** Trial sequential analysis of renal and urinary adverse events after combination therapy with PARP inhibitors and antiangiogenic drugs for ovarian cancer. (A) Urinary tract infection; (B) Proteinuria. Uppermost and lowermost red curves represent trial sequential monitoring boundary lines for benefit and harm, respectively. Horizontal green lines represent the conventional boundaries for statistical significance. Inner red lines represent the futility boundary.


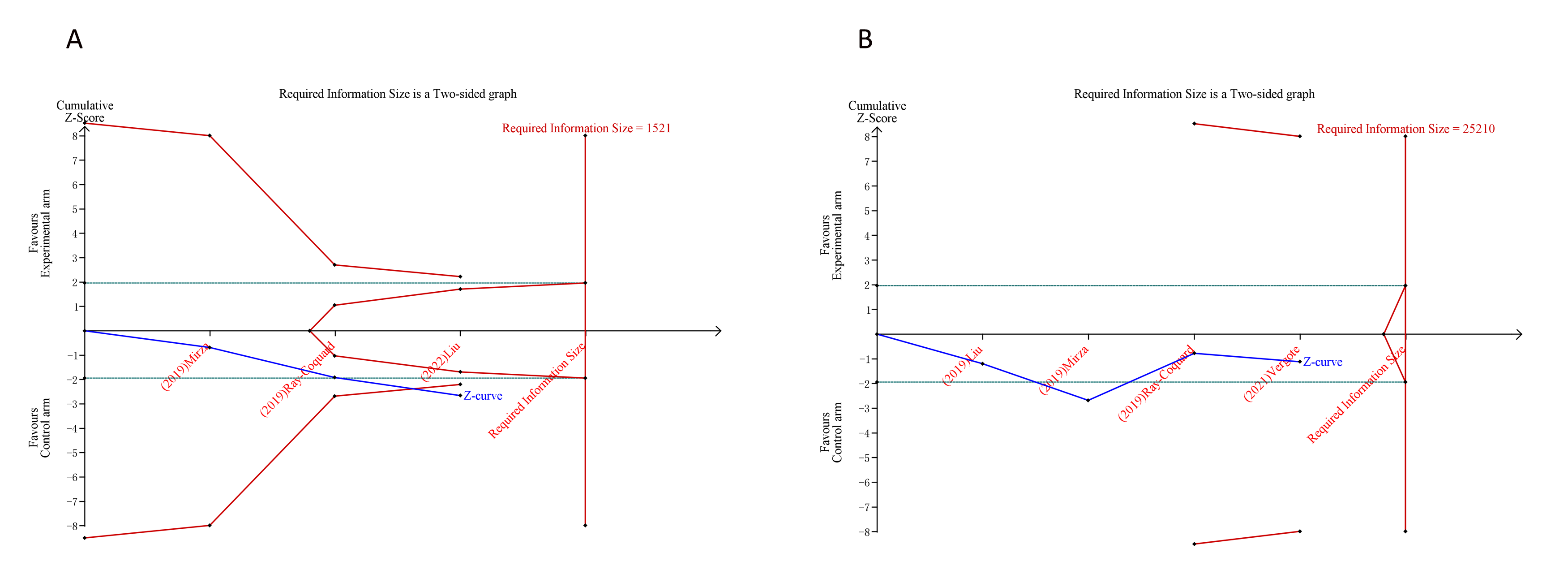


**FIGURE S11** Trial sequential analysis of other adverse events after combination therapy with PARP inhibitors and antiangiogenic drugs for ovarian cancer. (A) Fatigue; (B) Headache; (C) Anorexia; (D) Dyspnea; (E) Hypertension. Uppermost and lowermost red curves represent trial sequential monitoring boundary lines for benefit and harm, respectively. Horizontal green lines represent the conventional boundaries for statistical significance. Inner red lines represent the futility boundary.


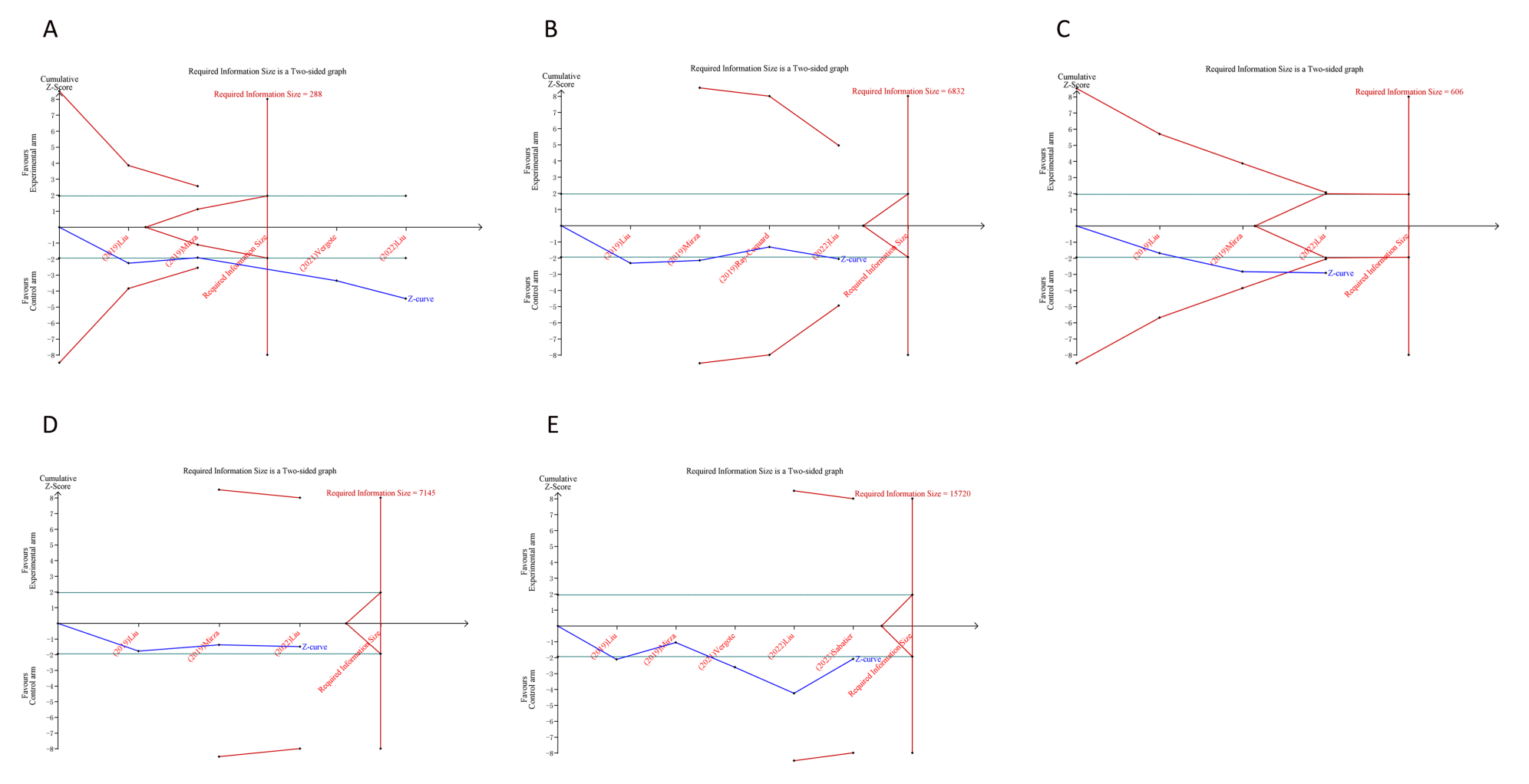

Supplement: Supplementary file 2 [file DataSheet2.docx]
