# Supplementary material for: Efficacy and safety of PARP inhibitors combined with antiangiogenic agents in the maintenance treatment of ovarian cancer: a systematic review and meta-analysis with trial sequential analysis of randomized controlled trials
Source: Front Pharmacol. 2024 Mar 22;15:1372077. doi: 10.3389/fphar.2024.1372077 (PMC10995238; doi:10.3389/fphar.2024.1372077)
Supplement: Supplementary file 3 [file DataSheet1.DOCX]

**PubMed 491**

#1 (angiogenesis inhibitor) OR (angiogenesis inhibitors) OR (tyrosine kinases inhibitors) OR (bevacizumab) OR (cediranib) OR (recentin) OR (avastin) OR (aflibercept) OR (Votrient) OR (sunitinib) OR (sorafenib) OR (zaltrap) OR (nexavar) OR (pazopanib) OR (trebananib) OR (perifosine) OR (nintedanib) OR (vargatef) OR (imatinib) OR (vandetanib) OR (AZD2171) OR (SU11248) OR (BIBF 1120) OR (AMG386) OR (BAY 545-9085) OR (BAY 43-9006) OR (AVE0005) OR (GW786034) OR (NSC-724772)

#2 (poly(ADP-ribose) polymerase inhibitor) OR (PARP inhibitor) OR (PARPi) OR (PARP inhibitors) OR (olaparib) OR (lynparza) OR (rucaparib) OR (talazoparib) OR (niraparib) OR (veliparib) OR (rubraca) OR (talzenna) OR (zejula) OR (AZD 2281) OR (AZD221) OR (ABT 888) OR (MK 4827) OR (AG014699) OR (BMN 673) OR (PF-01367338)

#3 ((ovar*) AND (cancer* OR neoplas* OR tumor* OR tumour* OR carcinoma* OR adenocarcinoma* OR malignan*)) OR ((cancer of ovary) OR (cancer of the ovary))

#4 #1 AND #2 AND #3

**Web of Science 755**

#1 TS=((angiogenesis inhibitor) OR (angiogenesis inhibitors) OR (tyrosine kinases inhibitors) OR (bevacizumab) OR (cediranib) OR (recentin) OR (avastin) OR (aflibercept) OR (Votrient) OR (sunitinib) OR (sorafenib) OR (zaltrap) OR (nexavar) OR (pazopanib) OR (trebananib) OR (perifosine) OR (nintedanib) OR (vargatef) OR (imatinib) OR (vandetanib) OR (AZD2171) OR (SU11248) OR (BIBF 1120) OR (AMG386) OR (BAY 545-9085) OR (BAY 43-9006) OR (AVE0005) OR (GW786034) OR (NSC-724772))

#2 TS=((poly(ADP-ribose) polymerase inhibitor) OR (PARP inhibitor) OR (PARPi) OR (PARP inhibitors) OR (olaparib) OR (lynparza) OR (rucaparib) OR (talazoparib) OR (niraparib) OR (veliparib) OR (rubraca) OR (talzenna) OR (zejula) OR (AZD 2281) OR (AZD221) OR (ABT 888) OR (MK 4827) OR (AG014699) OR (BMN 673) OR (PF-01367338))

#3 TS=(((ovar*) AND (cancer* OR neoplas* OR tumor* OR tumour* OR carcinoma* OR adenocarcinoma* OR malignan*)) OR ((cancer of ovary) OR (cancer of the ovary)))

#4 #1 AND #2 AND #3

**Embase 2383**

#1 'angiogenesis inhibitor'/exp OR 'angiogenesis inhibitors'/exp OR 'tyrosine kinases inhibitors' OR 'bevacizumab'/exp OR 'cediranib'/exp OR 'recentin'/exp OR 'avastin'/exp OR 'aflibercept'/exp OR 'votrient'/exp OR 'sunitinib'/exp OR 'sorafenib'/exp OR 'zaltrap'/exp OR 'nexavar'/exp OR 'pazopanib'/exp OR 'trebananib'/exp OR 'perifosine'/exp OR 'nintedanib'/exp OR 'vargatef'/exp OR 'imatinib'/exp OR 'vandetanib'/exp OR 'azd2171'/exp OR 'su11248'/exp OR 'bibf 1120'/exp OR 'amg386'/exp OR 'bay 545-9085' OR 'bay 43-9006'/exp OR 'ave0005'/exp OR 'gw786034'/exp OR 'nsc 724772'

#2 (poly AND 'adp ribose'/exp AND 'polymerase inhibitor'/exp OR 'parp inhibitor'/exp OR parpi) OR 'parp inhibitors' OR 'olaparib'/exp OR 'lynparza'/exp OR 'rucaparib'/exp OR 'talazoparib'/exp OR 'niraparib'/exp OR 'veliparib'/exp OR 'rubraca'/exp OR 'talzenna'/exp OR 'zejula'/exp OR 'azd 2281'/exp OR azd221 OR 'abt 888'/exp OR 'mk 4827'/exp OR 'ag014699'/exp OR 'bmn 673'/exp OR 'pf 01367338'/exp

#3 ovar* AND (cancer* OR neoplas* OR tumor* OR tumour* OR carcinoma* OR adenocarcinoma* OR malignan*) OR 'cancer of ovary' OR 'cancer of the ovary'

#4 #1 AND #2 AND #3

**The Cochrane Library 254**

#1 All Text=((angiogenesis inhibitor) OR (angiogenesis inhibitors) OR (tyrosine kinases inhibitors) OR (bevacizumab) OR (cediranib) OR (recentin) OR (avastin) OR (aflibercept) OR (Votrient) OR (sunitinib) OR (sorafenib) OR (zaltrap) OR (nexavar) OR (pazopanib) OR (trebananib) OR (perifosine) OR (nintedanib) OR (vargatef) OR (imatinib) OR (vandetanib) OR (AZD2171) OR (SU11248) OR (BIBF 1120) OR (AMG386) OR (BAY 545-9085) OR (BAY 43-9006) OR (AVE0005) OR (GW786034) OR (NSC-724772))

#2 All Text=((poly(ADP-ribose) polymerase inhibitor) OR (PARP inhibitor) OR (PARPi) OR (PARP inhibitors) OR (olaparib) OR (lynparza) OR (rucaparib) OR (talazoparib) OR (niraparib) OR (veliparib) OR (rubraca) OR (talzenna) OR (zejula) OR (AZD 2281) OR (AZD221) OR (ABT 888) OR (MK 4827) OR (AG014699) OR (BMN 673) OR (PF-01367338))

#3 All Text=(((ovar*) AND (cancer* OR neoplas* OR tumor* OR tumour* OR carcinoma* OR adenocarcinoma* OR malignan*)) OR ((cancer of ovary) OR (cancer of the ovary)))

#4 #1 AND #2 AND #3
